# Calcium signaling in postsynaptic mitochondria: mechanisms, dynamics, and role in ATP production

**DOI:** 10.3389/fnmol.2025.1621070

**Published:** 2025-07-21

**Authors:** Tatiana Feofilaktova, Liliia Kushnireva, Menahem Segal, Eduard Korkotian

**Affiliations:** ^1^Faculty of Biology, Perm State University, Perm, Russia; ^2^Department of Immunology and Regenerative Biology, The Weizmann Institute, Rehovot, Israel; ^3^Department of Brain Sciences, The Weizmann Institute, Rehovot, Israel; ^4^Faculty of Chemistry, Perm State University, Perm, Russia

**Keywords:** mitochondria, mitophagy, dendritic spine, ATP synthase, endoplasmic reticulum, MCU, MAM, Ca^2+^ signaling

## Abstract

While the overall ATP level in neurons remains relatively stable, local fluctuations in synaptic compartments - driven by synaptic potentials - necessitate rapid ATP adjustments. The energy supply for synaptic activity in neurons must be under precise homeostatic control: increased ATP consumption in active synapses requires continuous replenishment, whereas in periods of inactivity, excess ATP production may occur. Overproduction of ATP in thousands of individual synapses is metabolically wasteful, while underproduction threatens to disrupt molecular cascades associated with ongoing synaptic bursts, ion homeostasis, protein synthesis, and neural plasticity. Fine-tuned regulation of ATP synthesis must therefore be controlled locally and dynamically, ensuring metabolic efficiency while preventing disruptions in synaptic bursts, ion homeostasis, and neuronal plasticity. This review summarizes the intricate molecular mechanisms through which mitochondria (MT) interact with their postsynaptic environment to maintain energy balance. We examined the fundamental features of mitochondria in conjunction with their unique properties and roles in nervous tissue, highlighting their ability to dynamically adjust energy production based on local demand rather than maintaining a strictly uniform ATP output. The regulation of ATP synthesis may involve mitochondrial transport, fusion, and fission, as well as changes in mitochondrial shape and molecular structure. This review describes the activity of ATP synthase, the mitochondrial calcium uniporter and other signaling cascades in the context of their uneven distribution within mitochondria. Furthermore, we discuss rapid calcium influxes from postsynaptic membranes and the endoplasmic reticulum into mitochondria-associated membranes (MAMs), their buffering mechanisms, and the generation of dynamic responses. We focus on the role of calcium ion (Ca^2+^) as a precise regulator of ATP production, particularly in mitochondria located near synaptic regions, where it ensures an adequate energy supply for local activity. Overall, we propose potential pathways of interaction between mitochondria and their postsynaptic microdomains. Given that some of the mechanisms discussed remain hypothetical, we emphasize the urgent need for experimental validation to refine understanding of mitochondrial function in synaptic transmission.

## 1 Introduction

Energy is a fundamental necessity for all cells, enabling their functional activity, growth, and survival. Neurons, however, differ significantly from other cell types due to their unique characteristics: they do not undergo cell division and possess exceptionally long processes—axons and dendrites—that extend across considerable distances to facilitate complex communication networks. This structural specialization demands substantial energy, particularly at synaptic sites where neurotransmission occurs. The primary source of cellular energy is adenosine triphosphate (ATP), which is predominantly synthesized by mitochondria (MT)—symbiotic organelles present in most eukaryotic cells. MT perform multiple functions, some of which remain incompletely understood. Their energy-producing role is closely tied to ATP synthases, which facilitate ATP generation by harnessing proton gradients across mitochondrial membranes. However, mitochondrial functions are dynamic and depend on their structural organization, which undergoes continuous remodeling in response to physiological changes. These alterations affect mitochondrial transport, docking, fusion, and fission (McCarron et al., 2013; Trigo et al., 2022). Notably, elongated MT enhance oxidative capabilities, whereas spherical or ovoid forms are often associated with increased calcium (Ca^2+^) levels in cytosol, adaptive mechanisms, or pathology (Glancy et al., 2020).

Beyond mitochondrial ATP synthesis, additional mechanisms contribute to maintaining energy homeostasis. Membrane-associated ATP synthases can support localized ATP production, ensuring energy availability in specific subcellular compartments (Xing et al., 2011; Chang et al., 2023). Astrocytes also play a crucial role in metabolic support through the astrocyte-neuron lactate shuttle, a mechanism that links astrocytic glycolysis to neuronal oxidative metabolism (Chih and Roberts, 2003; Roumes et al., 2023). Particularly, such shuttle mechanism may appear during memory formation (Drulis-Fajdasz et al., 2018) and some forms of synaptic plasticity (Marty-Lombardi et al., 2024; Kim et al., 2025).

Cytosolic ([Ca^2+^]_*c*_) and mitochondrial ([Ca^2+^]_*m*_) calcium signaling play a critical role in regulating ATP synthesis and cellular energy balance. The voltage-dependent anion channel (VDAC) in the outer mitochondrial membrane (OMM) serves as the primary gateway for Ca^2+^ entry into the MT from the cytosol (Rajendran et al., 2023). Once inside, Ca^2+^ transport is facilitated by the mitochondrial calcium uniporter (MCU) complex, which governs mitochondrial Ca^2+^ uptake and influences enzymatic activity within the tricarboxylic acid cycle (TCA, also known as the Krebs cycle), thereby enhancing ATP production (De Mario et al., 2023). While this mechanism is particularly vital in neurons, it also operates in highly active cells such as cardiac myocytes and endocrine cells, where precise control of energy metabolism is essential for physiological function. Additionally, ATP synthase (F-ATPase), responsible for ATP production, undergoes conformational changes driven by the proton gradient, with its rotor-stator architecture optimizing energy conversion. Tertiary structures of MCU, VDAC and ATP synthase have been resolved and successfully used for structure-function link (see for review: Najbauer et al., 2021; Zhuo et al., 2021; Vlasov et al., 2022).

Ca^2+^ directly modulates ATP synthase activity by influencing its conformational states, potentially enhancing enzyme efficiency and mitochondrial energy output. However, disruptions in [Ca^2+^]_*m*_ handling are implicated in various pathological conditions, including neurodegenerative diseases, such as Alzheimer’s and Parkinson’s disease, cardiovascular disorders, and metabolic syndromes (see for review: Walters and Usachev, 2023; Balderas et al., 2024; Zong et al., 2024; Borbolis et al., 2025; Sun et al., 2025). Dysregulated Ca^2+^ uptake through the VDAC-MCU complex can lead to mitochondrial dysfunction, excessive oxidative stress, and even apoptosis, highlighting the need for maintaining [Ca^2+^]_*m*_ homeostasis for overall cellular health (Giorgio et al., 2017; Wang et al., 2025).

In neurons, Ca^2+^ signaling mechanisms and alternative energy supply pathways are particularly crucial in the context of postsynaptic mitochondria in neurons, where fluctuations in energy demands closely interact with neuronal activity. Ca^2+^ dynamics regulate mitochondrial function by influencing ATP synthesis and metabolic coupling, ensuring that synaptic compartments maintain adequate energy levels for neurotransmission and plasticity (Groten and MacVicar, 2022). Additionally, Ca^2+^ plays a key role in shaping mitochondrial distribution, morphology, ion balance, and functional modulation under both normal and pathological conditions. This review will explore these aspects in detail, highlighting their significance in cellular health and disease.

## 2 Morphology and ultrastructure of mitochondria

### 2.1 Mitochondrial morphologies in different cell types

**FIGURE 1:**
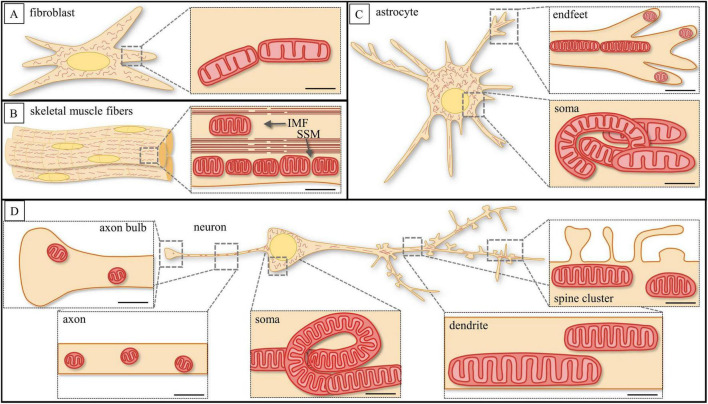
Schematic illustration of mitochondrial morphological variations across cell types and subcellular compartments. **(A)** Mitochondria (MT) of fibroblasts have elongated form with less density of cristae arrangement. (**B**) MT of skeletal muscle fibers have small and short sizes with high density of cristae arrangement. The MT categorized into two populations: intermyofibrillar (IMF) mitochondria located between myofibrils and subsarcolemmal (SSM) mitochondria clustered beneath the plasma membrane. **(C)** MT of astrocytes have diverse morphological properties; MT of astrocytic somata have elongated, twisted form with less density of cristae arrangement, whereas MT of astrocytic endfeet have small, elongated thin structure under endfeet base. (**D**) MT of neurons have great diversity of size and shape; MT of the axon and the axon bulb have small size and short and globular form with high density of cristae arrangement. MT of neuron’s soma have elongated, twisted form with less density of cristae arrangement. MT of dendrites and within spine cluster have elongated form with less density of cristae arrangement. Scale bar: 1 μm. References to electron microscope images of MT in fibroblasts (Brantová et al., 2006), skeletal muscle cells (Wahwah et al., 2020), astrocytes (Pysh and Khan, 1972; Bergami and Motori, 2020), pyramidal neurons (Faitg et al., 2021).

MT exhibit diverse morphologies and mobility, ranging from spherical to elongated structures, with their distribution and movement regulated according to cell type and metabolic requirements (Figure 1; Table 1). MT size ranges from 0.5 to more than 2 μm, in cross section (Frey and Mannella, 2000; Perkins and Frey, 2000). Their morphology and distribution vary significantly depending on cellular function. Thus, in fibroblasts, mitochondria are approximately the same size and dispersed evenly, supporting anabolic processes (Figure 1A; Brantová et al., 2006), whereas in skeletal muscle cells, they are categorized into two main populations: intermyofibrillar (IMF) mitochondria, which are located between myofibrils and primarily support oxidative metabolism, and subsarcolemmal (SSM) mitochondria, which cluster beneath the plasma membrane and are more involved in localized energy supply (Figure 1B; Wahwah et al., 2020; Pekkurnaz and Wang, 2022). The SSM mitochondria tend to align in chain-like structures, optimizing ATP distribution for membrane-associated functions and signaling processes (Holmuhamedov et al., 2012). In brain tissue, the morphological differences between MT in neurons and glial cells become increasingly complex depending on the cellular compartment. Thus, in astrocytes, MT are relatively abundant within the soma, where they display diverse orientations, supporting the cell’s metabolic and regulatory functions. As astrocytic processes extend and thin, mitochondrial morphology transitions from a more branched and disorganized network in the soma to elongated, parallel structures within finer outgrowths, particularly in perivascular endfeet, where they often appear more compact and fragmented, likely reflecting localized energy demands and dynamic Ca^2+^ signaling (Figure 1C; Popov et al., 2023; Salazar et al., 2024). In neurons, MT exhibit the highest degree of compartmental specialization, adapting their morphology and dynamics to meet distinct metabolic requirements: in the soma, mitochondria form elongated, interconnected networks with diverse orientations, while in dendrites, mitochondria are more linear and tubular, with some exceeding 1 μm^3^ in volume, whereas shorter forms are occasionally observed within dendritic spines. In axons, mitochondria are highly mobile, typically punctate, and rarely surpass 1 μm^3^, ensuring efficient energy distribution along thin but exceptionally long axonal projections (Figure 1D; Faitg et al., 2021; Pekkurnaz and Wang, 2022). The heterogeneity in mitochondrial volume reflects the overall complexity of the cell, with highly differentiated cells requiring precise spatial and morphological adaptations.

**TABLE 1:** Comparison of some mitochondrial characteristics in different cell types.

Cell type		MT size, μm	MT membrane potential (ΔΨmt ), mV	Speed of movement (anterograde), μm/s)	Speed of movement (retrograde), μm/s
Neurons	Axons	∼1.4 (Chang et al., 2006)	–108 to –158 (Gerencser et al., 2012; Zorova et al., 2018)	∼ 0.4–0.6 (Ligon and Steward, 2000; Niescier et al., 2016)	∼ 0.5 (Ligon and Steward, 2000; Niescier et al., 2016)
	Dendrites	∼ 2.2 (Chang et al., 2006)		∼ 0.5 (Ligon and Steward, 2000)	∼ 0.4 (Niescier et al., 2016)
	Soma	0.72 — 3.56 (Narayanareddy et al., 2014)		∼ 0.8 (Narayanareddy et al., 2014)	
Astrocytes		∼ 2.4 (Stephen et al., 2015)	∼ –150 (Feeney et al., 2003)	∼ 0.15 (Jackson and Robinson, 2018)	∼ 0.2 (Jackson and Robinson, 2018)
Cardiomyocytes		∼ 1–2 (Li et al., 2023)	∼–140 to –150 (Di Lisa et al., 1995; Mathur et al., 2000)	Near nuclear: 0.05 Near periphery: 0.005 (Kassab et al., 2021)	
Fibroblasts		∼ 1 (Nadalutti and Wilson, 2020; Goldstein et al., 1984)	∼ –91 to –112 (Gurm et al., 2012; Huang et al., 2004)	∼ 0.35 (Drabik et al., 2021)	
Skeletal muscle fibers		∼ 0.2 μm^2^ (Picard et al., 2013)	∼ –147 (Pelletier-Galarneau et al., 2017)	∼ 0 (around 0.0008) (Iqbal and Hood, 2014)	

### 2.2 Mitochondrial ultrastructure

**FIGURE 2:**
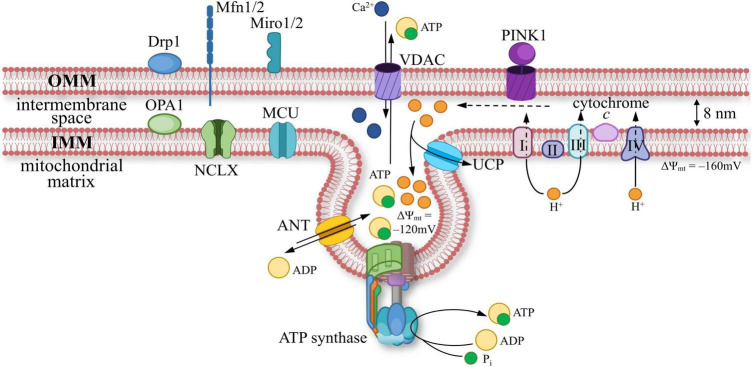
A scheme of mitochondrial membrane ultrastructure: components of the outer (OMM) and inner mitochondrial membrane (IMM). Proteins present in OMM: dynamin-related protein 1 (Drp1, responsible for mitochondrial fission); dynamin-like GTPase mitofusins 1/2 (Mfn1/2, facilitate physical connections between OMM and other membranes); mitochondrial Rho GTPase 1/2 (Miro1/2, acts as a calcium (Ca^2+^) sensor; PINK1 (PTEN-induced kinase 1, initiates mitophagy); voltage-dependent anion channel (VDAC, responsible for the transport of ions and nucleotides). IMM is equipped with proteins such as: dynamin-like GTPase optic atrophy 1 (OPA1, facilitates mitochondrial fusion); electron transport chain (ETC) components, includes complexes I, II, III, IV, and cytochrome c; ATP synthase (catalyzes the synthesis of ATP), adenine nucleotide translocator (ANT, enables ADP/ATP exchange); mitochondrial calcium uniporter (MCU, facilitates Ca^2+^ transport); uncoupling protein (UCP, capable of dissipating the proton gradient); Na^+^/Ca^2+^ exchanger (NCLX).

At the ultrastructural level, MT are surrounded by two phospholipid membranes with massive protein insertions (Figure 2). OMM is abundantly equipped with integral beta-barrel channels or porins to transport hydrophilic molecules. These porins include the VDAC, which is the most abundant protein on OMM and is involved in the transport of ATP and ADP anions, Ca^2+^, and other metabolites, as well as playing a key role as a switch in mitochondrial functions (Rostovtseva and Bezrukov, 2008; Noskov et al., 2013; Rajendran et al., 2023). There is an intermembrane space (∼ 8 nm) between OMM and the inner mitochondrial membrane (IMM) (Neupert, 1997). The ion concentration within this space is similar to that of the cytosol. In contrast, the IMM provides a high level of impermeability, which is crucial for maintaining a stable proton gradient across the membrane. The impermeability of the IMM is primarily provided by the phospholipids cardiolipin and phosphatidylethanolamine, which have double hydrophobic “tails” of fatty acids (Ikon and Ryan, 2017). The proton gradient is maintained by the energy derived from aerobic respiration, which is a system of redox reactions in transmembrane protein complexes called electron-transport chain (ETC). Additionally, there are transmembrane proteins in the IMM that support metabolic connections with the space within IMM, called the matrix, including a critically important protein that mediates the entry of Ca^2+^ into the matrix—MCU. The IMM has a highly folded structure in the form of creases called cristae to increase the surface area. The ionic and molecular composition of the narrow space between the cristae may differ compared to the rest of the matrix.

### 2.3 Structure and location of mitochondrial ATP synthase

**FIGURE 3:**
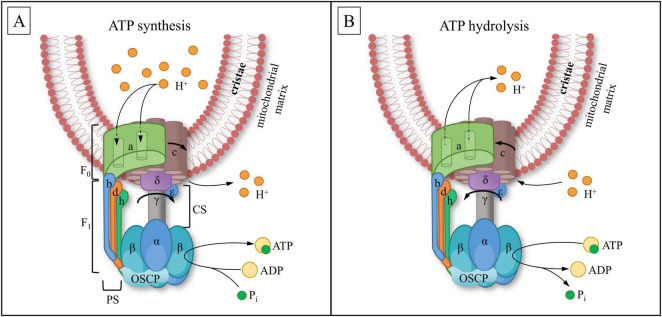
A scheme of mitochondrial ATP synthase composition during ATP synthesis **(A)** and hydrolysis. **(B)** ATP synthase converts the energy of the proton electrochemical transmembrane gradient into ATP through mechanical rotation. F_1_ complex is a hexamer composed of α- and β-subunits, connected to the peripheral stalk (PS), which includes b-, d-,and h (F6 in mammals)-subunits, and the central stalk (CS), containing γ-, ε-, and δ-subunits. F_0_ is a transmembrane complex composed of the c-ring and the a-subunit. OSCP (oligomycin-sensitivity conferring protein) — connects F_1_ with PS, provides structural stability and coupling the rotary action of the F_0_ domain to ATP synthesis in F_1_. **(A)** The proton electrochemical gradient (H^+^) drives the rotation of the c-ring within the F_0_ complex. This motion initiates conformational changes in the β-subunits of the F_1_ complex, leading to the sequential binding of ADP and inorganic phosphate (Pi) and ATP release. **(B)** In conditions of low membrane potential, ATP is utilized to drive the reverse rotation of the c-ring within the F_0_ complex. This motion initiates conformational changes in the β-subunits of the F_1_ complex, leading to sequential ATP cleavage into ADP and inorganic phosphate (Pi), leading to proton translocation across the membrane.

ATP molecules are synthesized in the mitochondrial matrix from ADP and inorganic phosphate by using electrochemical energy produced by the proton gradient (Neupane et al., 2019; Mnatsakanyan and Jonas, 2020b). The process of ATP synthesis is generated by a specific enzyme, also anchored in the IMM, known as ATP synthase. In a broad sense, ATP synthase is related to the superfamily of rotary ATPases. Rotary ATPases can catalyze ATP hydrolysis to perform useful work, such as ion transfer across cellular membranes. Conversely, they can synthesize ATP through directed ion flow, more specifically via proton leakage along the concentration gradient, while protons re-enter the mitochondrial matrix through ATP synthase (Watson and McStay, 2020). Thus, ATP synthase can operate in both directions—synthesizing ATP or hydrolyzing it—depending on the current local needs of the cell (Figure 3). Eukaryotic ATPases are divided into two types: F-ATPase and V-ATPase (Jonckheere et al., 2012). F-ATPase has the ability to synthesize and hydrolyze ATP (Jonckheere et al., 2012; Pinke et al., 2020). Nevertheless, in MT, F-ATPase primarily functions as an ATP synthase. In contrast, V-ATPase exclusively hydrolyzes ATP to obtain energy for proton pumping across membranes. V-ATPase is present not only in the IMM, but also in various cellular membranes, where it is essential for the acidification of endosomes, lysosomes, and the trans-Golgi network (Abbas et al., 2020; Zubareva et al., 2020).

The composition of F-ATPase is presented in Figures 3A,B. It consists of two components: the transmembrane proton pore F_0_ and the inner component F_1_. The hexamer structure of F_1_ consists of α- and β-subunits, which alternate with each other and surround the γ-subunit. The central stalk (CS) and the peripheral stalk (PS) connect through the δ and ε subunits and oligomycin-sensitivity conferring protein (OSCP), b, d, and h (F6 in mammals) subunits (Walker and Dickson, 2006; Gerle, 2020). Proton transfer into the matrix is achieved through the rotation of the c-ring composed of several c-subunits and the a-subunit, which contains a proton channel, all coupled with the rotation of the γ-subunit. The CS passes through the center of the F_1_ hexamer, connecting the transmembrane part of F_0_ with the catalytic part of F_1_. The PS acts as a stator, preventing the co-rotation of the F_1_ domain (Giorgio et al., 2017; Gerle, 2020). The height of the transmembrane domain F_0_ reaches ∼6.4 nm and the diameter of ∼10–12 nm (Hatefi, 1993). The F_1_ reaches a length of ∼11–12 nm and a diameter of ∼7.4 nm, the CS has length of approximately ∼4.3–4.5 nm (Hatefi, 1993; Nirody et al., 2020; Nesterov and Yaguzhinsky, 2023). The F_1_ domain plunges into mitochondrial matrix due to its hydrophilic properties. Specifically, this domain is responsible for the synthesis and/or hydrolysis of ATP (Walker and Dickson, 2006; Nirody et al., 2020; Frasch et al., 2022).

As discussed above, although the eukaryotic F-ATPase (a member of the ATPase family) is typically associated with ATP synthesis, it can work in opposite directions: using the proton gradient for synthesis (Figure 3A) or hydrolyzing ATP to create a proton gradient (Figure 3B).

ATP production is achieved by the transmembrane electrochemical proton gradient generated by the ETC, resulting in the mitochondrial membrane potential (ΔΨ_mt_). The threshold level of ΔΨ_mt_ in IMM required to initiate the rotation of ATP synthase’s rotor is –65 mV in mammals (Wescott et al., 2019). The value of ΔΨ_mt_ can vary up to –170 mV, with the rate of ATP synthesis increasing nonlinearly with increasing potential (Wescott et al., 2019; Kaim and Dimroth, 1999). Effective ATP generation occurs when ΔΨ_mt_ is around –100 to –120 mV (Lee et al., 2002). In addition to the transmembrane electrochemical proton gradient (ΔΨ_mt_), the reduced form of NADH (TCA product) is required for ATP generation (Meléndez-Hevia et al., 1996; Sonenshein, 2001; Bonora et al., 2012). Some of the TCA dehydrogenases are Ca^2+^-dependent enzymes. Therefore, activation of these dehydrogenases requires Ca^2+^ influx into mitochondria and the presence of a Ca^2+^ buffer (to be discussed later) (Bonora et al., 2012; Markovinovic et al., 2022).

The maintenance of ΔΨ_mt_, ETC, and ATP synthase function is supported by several key messengers and factors that are universal in eukaryotic cells. Among these are the ATPase Inhibitory Factor 1 (IF1) (García-Bermúdez and Cuezva, 2016; Glancy and Balaban, 2012; Gore et al., 2022) and Ca^2+^ (Glancy and Balaban, 2012; Wescott et al., 2019), which regulate mitochondrial bioenergetics, and nitric oxide (NO) produced by nitric oxide synthase (NOS) within the IMM (Finocchietto et al., 2009; Kohlhaas et al., 2017; Godoy et al., 2021). In neuronal cells in particular Ca^2+^ can enter the mitochondria from the extracellular space via various pathways, such as activation of N-methyl-D-aspartate receptors (NMDARs), a subtype of glutamate receptors (Garthwaite, 2008; Negri et al., 2021), highlighting the specialized role of these receptors in regulating mitochondrial function in the postsynaptic region, opening of voltage-gated calcium channels, engaging store-operated calcium currents, and activating other pathways (see for review: Nanou and Catterall, 2018; Chin and Kaeser, 2024). We will discuss this issue in more detail in section 6.

## 3 Mitochondria in central neurons

### 3.1 Features of neuronal mitochondria

Despite the similarities in physiology and functional principles of mitochondria across all tissue types, there are unique characteristics and specific patterns of mitochondrial structure, dynamics, form and localization that are tailored to the energy demands and functions of specific cell types. In the central nervous system tissue, there are several cell types, including neurons and glial cells such as astrocytes, microglia, and oligodendrocytes (Garman, 2011; Herculano-Houzel, 2014; Liu et al., 2023). In comparison with glial cells (Kann and Kovács, 2007; Jackson and Robinson, 2018), cardiomyocytes and skeletal muscle fibers (Kuznetsov et al., 2009), neuronal MT are more motile in both ratio (for instance, the number of mobile MTs in neurons is approximately twice as high as in glia (Jackson and Robinson, 2018) and velocity of the motion: ∼0.3 μm/s in neurons and ∼0.1 μm/s in astrocytes (Stephen et al., 2015; Table 1). Moreover, the speed of mitochondrial movement in neurons and astrocytes varies depending on the direction, either anterograde (from the soma) or retrograde (toward the soma), but the speed of neuronal MT is around three times greater than astrocytes’ one (Jackson et al., 2014; Table 1). Furthermore, in comparison with astrocytes, dysfunctional mitochondria elimination is more developed in neurons (Sukhorukov et al., 2021), which may impact the functionality of these cells (see in the next chapter). MT features within different types of neurons also possess some differences. As an example, MT of fast-spiking [∼ 30–100 Hz (Kann et al., 2014; Kann, 2016)] inhibitory interneurons are distinct from MT of cortical neurons. The unique physiological properties of interneuronal MT require the enhancement of ETC in the IMM, enriched in a number of proteins such as cytochrome c oxidase and the ETC complex I (Whittaker et al., 2011; Kann et al., 2014; Kann, 2016) which ensure the dynamics of ATP synthase and cell survival. As for MT velocity in the neurons of different origins, it may be affected by multiple factors, for example, by activity, stages of cell development (Silva et al., 2021) or by activating modulatory pathways (Pekkurnaz et al., 2014; Jenkins et al., 2024), for example by the AKT–glycogen synthase kinase 3β pathway in serotoninergic and dopaminergic neurons (Pekkurnaz and Wang, 2022).

Little is known about ΔΨ_mt_ in neurons, which differ in morphology, origin, and the main transmitter released. One study showed that dopaminergic cells of the substantia nigra keep relatively low ΔΨ_mt_, ∼ –94 mV (Huang et al., 2004). Higher values were found in several types of cultured neurons: –100 mV in hippocampal culture (Korkotian et al., 2019), –139 mV in cortical neurons (Gerencser et al., 2012) and –150 mV in cerebellar granule cells (Ward et al., 2000). Several physiological factors, including depolarization, hyperpolarization and overall activity are believed to influence ΔΨ_mt_, contributing to its volatility and dynamic instability. In general, it should be noted that neuronal ΔΨ_mt_ fluctuates within a fairly wide range, comparable to the potential in astrocytes and myocytes, but exceeding it in fibroblasts (Table 1).

Thus, neuronal and especially dendritic MT possess a set of unique features that shape their specialized metabolic properties, which will be explored in the context of Ca^2+^ buffering and ATP production in the following sections.

### 3.2 Mechanisms of mitochondria motility in neurons

**FIGURE 4:**
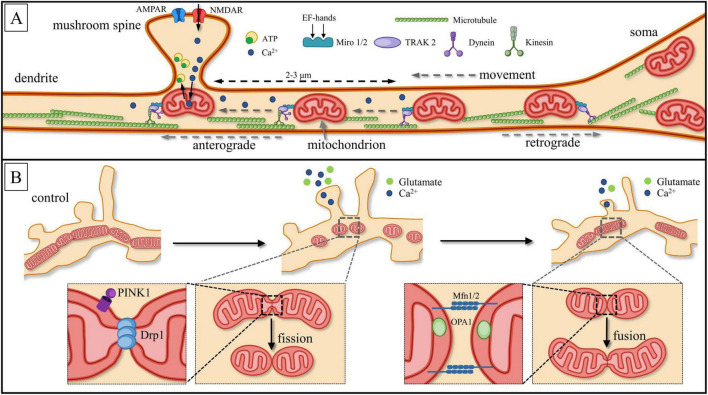
Schematic description of neuronal mitochondria motility. **(A)** Miro1/2 (Mitochondrial Rho GTPase1/2) acts as a Ca^2+^ sensor. Through binding with the motor-adaptor protein TRAK2, Miro1/2 inhibits the interaction between dynein and microtubules. This inhibition allows mitochondria to remain within the local Ca^2+^ burst region (dotted line), which occurs due to the activation of N-methyl-D-aspartate receptors (NMDARs). The presence of four EF-hand domains in Miro1/2 facilitates this process. Local Ca^2+^ bursts are restricted to ∼2–3 μm from the site of origin (dashed line). As a result, mitochondria are anchored beneath the base of dendritic spines, optimizing ATP production and enabling efficient Ca^2+^ buffering. Different motor proteins provide movement in anterograde (toward the terminals) or retrograde (toward to the soma) directions: kinesin provides anterograde transport, while dynein provides retrograde transport. **(B)** Another form of mitochondrial motility involves mitochondrial “fission,” which separates organelles, and “fusion,” where they merge into a single cluster. Fission is regulated by the GTPase Dynamin-1-like protein, Drp1, while mitochondrial fusion is controlled by Mitofusins (Mfn) and the dynamin-like GTPase Optic Atrophy 1 (OPA1). During local synaptic glutamate release, high concentrations of [Ca^2+^]_c_ can induce mitochondrial fission, whereas low concentrations of [Ca^2+^]_c_ promote fusion. This scheme is based on unpublished confocal images generated by the authors.

As mentioned above, neuron is a type of cell that exhibits a spatially polarized and elongated form. The MT structure, clustering, and distribution in neurons are highly distinct due to the differentiation of neuronal compartments (Perkins et al., 2001; Kann and Kovács, 2007) and the significant length of their processes, which can extend several meters from the cell soma (Cai and Sheng, 2009a). Neuronal MT mobility occurs through their movement along cytoskeletal elements such as microtubules and actin filaments (Shah et al., 2021; Alberti et al., 2022; Zaninello and Bean, 2023). There is a specific principle for microtubule orientation in neurons: in axons and distal dendrites, microtubules are strictly oriented such that their dynamic ends, or plus-ends, are conditionally directed toward the terminals, while their stable ends, or minus-ends, are directed toward the cell soma. Different motor proteins provide movement in anterograde (toward the terminals) or retrograde (toward the soma) directions (Figure 4A). Thus, the superfamily of kinesin provides mostly for anterograde transport, while dynein provides retrograde transport. In proximal dendrites, the orientation of microtubules could be mixed, with both minus- and plus- ends present, so the direction of motility in dendrites is not solely dependent on motor proteins (Hirokawa and Takemura, 2005; Kapitein et al., 2010; Kruppa and Buss, 2021). In the neuronal soma, mitochondria exhibit specific orientations related to the microtubule network, contributing to the overall cellular architecture and function (Petersen et al., 2014; Seager et al., 2020).

Mitochondria are attached to motor proteins through specific adaptor proteins such as Miro1/2 and TRAK1 (Trafficking Kinesin Protein 1) and TRAK2, both homologues of Milton (MacAskill et al., 2010; Misgeld and Schwarz, 2017; Kruppa and Buss, 2021; Duarte et al., 2023). TRAK1 is responsible for axonal transport and binds with both kinesin and dynein, while TRAK2 binds exclusively with dynein, promoting dendritic mitochondrial transport (Sheng, 2014; Seager et al., 2020). It was established that Miro1/2 works not only as GTPases, acting as molecular switchers by binding and hydrolyzing guanosine triphosphatases (GTP) but is also Ca^2+^ sensitive (Cai and Sheng, 2009b; Lee et al., 2018; Duarte et al., 2023). Miro EF-hands act as Ca^2+^ sensors and inhibit kinesin, leading to the inactivation of the Miro-TRAK-kinesin complex.

Local Ca^2+^ gradients play an essential, though not fully understood, role in the mitochondrial movement. On the one hand, the increase in [Ca^2+^]_*c*_ in the active zones should attract MT, causing their movement toward the activation site, specifically to the presynaptic bouton and/or the active spine. On the other hand, the movement of MT should halt due to the rise in [Ca^2+^]_*c*_ concentration at the activation site. The Ca^2+^ sensors are in the OMM whereby mitochondria connect not only with motor protein but move along microtubules towards local Ca^2+^ gradients caused by ongoing synaptic activity (MacAskill et al., 2010; Lin and Sheng, 2015; Rangaraju et al., 2019b; Kushnireva and Korkotian, 2022). Neuronal activity, cytosolic calcium rises, and synaptic glutamate release contribute to a reduction of speed of mitochondrial movement (MacAskill et al., 2009). The growth of synaptic activity reduces dendritic mitochondrial mobility along microtubules and enhances their density at the spine base, especially in mushroom-type spines, where mitochondria organize into stable tubular elongated clusters (Lee et al., 2018; Thomas et al., 2023). Inhibiting the connection between mitochondria and motor kinesin within the mitochondrial motor-adaptor complex leads to the undocking of the mobile fraction of MT from microtubules and their docking in areas of local [Ca^2+^]_*c*_ spikes (Figure 4A; Sheng, 2014; Duarte et al., 2023). It is assumed that Ca^2+^-induced mitochondrial transport arrest near active synapses provides alternate energy to glutamatergic synapses (Colgan and Yasuda, 2014; Duarte et al., 2023).

The largest difference in MT shape and dynamics is observed between axonal and dendritic compartments, favoring larger sizes (with some exceptions) and slower dynamics for the latter. For example, the volume of dendritic mitochondria exceeds that of axons and soma in the dentate gyrus of the hippocampus, but not in the CA1 region (Faitg et al., 2021). Also, layer 2/3 of cortical neurons exhibit elongated mitochondrial morphology in dendrites (range from 1.31 to 13.28 μm) compared to axonal MT (ranging from 0.45 to 1.13 μm), both *in vivo* and *in vitro* (Lewis et al., 2018; Seager et al., 2020; Yao et al., 2020). Axonal mitochondria exhibit greater velocity and longer-range motility and a generally globular or ovoid shape (Seager et al., 2020; Chang et al., 2006). In dendrites, mitochondria exist as extended tubular clusters with high density (Li et al., 2004). A particularly high density of mitochondria is observed in postsynaptic areas, where it supplies local protein synthesis by ribosomal complex, support ionic, or more precisely Ca^2+^ homeostasis and cytoskeleton structural re-modeling (Chang et al., 2006; Palikaras and Tavernarakis, 2020; Murali Mahadevan et al., 2021; Duarte et al., 2023; Thomas et al., 2023; Bapat et al., 2024). Occasionally, postsynaptic MT can invade dendritic spine head (Bosch and Hayashi, 2012; Thomas et al., 2023), however, typically it is concentrated in the parent dendrites under the spine neck (Nimchinsky et al., 2002; Chang et al., 2006; Bourne and Harris, 2008; von Bohlen Und Halbach, 2009; Thomas et al., 2023).

Furthermore, greater mobility is typical of newly formed mitochondria, created by fission (see below). This MT pool maintains high mobility within the transport motor/adaptor complex due to its small size (Gu et al., 1994; Sheng and Cai, 2012; Segal and Korkotian, 2014; Green et al., 2022). It has been shown that this complex moves directly to areas of elevated [Ca^2+^]_*c*_ concentration (Colgan and Yasuda, 2014; Duarte et al., 2023).

### 3.3 Mechanisms of mitochondria motility in dendrites

It is generally assumed that synaptic activity determines the topography and dynamics of mitochondria associated with the relevant dendritic spines, as well as the total length of mitochondrial clusters (Bereiter-Hahn and Jendrach, 2010; Leung et al., 2021; Thomas et al., 2023). This depends on two types of opposing processes: cluster division or “fission” to separate organelles and merging or “fusion” into one cluster (Figure 4B). Fission is controlled by GTPase from the dynamin superfamily: DNM1L (Dynamin-1-like protein) or Drp1 (dynamin-related protein 1) (Pekkurnaz and Wang, 2022; Duarte et al., 2023). Disruption or delay in fission caused by Drp1 dysfunction prevents mitophagy of damaged mitochondria, which negatively impacts synaptic function. Drp1-dependent modulation of anti-apoptotic proteins Blc-w and Blc-xL expression exerts influence on MT location and is correlated with an increase in protein number in the postsynaptic density (MacAskill et al., 2010). Drp1-dependent mitochondrial division has been increased by the influence of PTEN-induced kinase 1 (Phosphatase and tensin homolog PINK1) and Ca^2+^ leak following synaptic potentiation (Duarte et al., 2023; Thomas et al., 2023). Enhanced mitochondrial fission promotes structural morphological alterations of dendritic spines in turn (Duarte et al., 2023).

Mitofusins (Mfn) and the dynamin-like GTPase Optic Atrophy 1 (OPA1), which facilitate mitochondrial fusion, play a crucial role in stabilizing mitochondrial morphology (Williams et al., 2010; Emery and Ortiz, 2021). Their dysfunction can result in pathological fragmentation of the organelle and various degenerative conditions (Pellegrini and Scorrano, 2007; Knott and Bossy-Wetzel, 2008; Santos et al., 2015). The mutual and balanced work of Mfn2 and Drp1 is required to maintain physiological function and balance between fission and fusion (MacAskill et al., 2010; Misgeld and Schwarz, 2017). Moreover, the proteins’ connection with transport proteins such as Miro and TRAK are necessary to maintain different forms of synaptic plasticity (MacAskill et al., 2010; Eberhardt et al., 2020).

**FIGURE 5:**
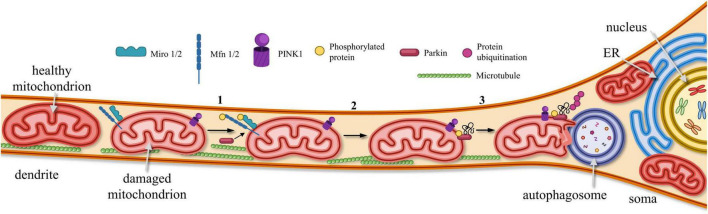
Elimination of dysfunctional MTs from postsynaptic sites. In dendrites, dysfunctional MTs are removed via PINK1-Parkin signaling pathway: (1) PINK1 accumulates on OMM, promoting phosphorylation of Miro1/2, mitofusin proteins (Mfn1/2) and Parkin. (2) Phosphorylated Parkin becomes anchored to OMM, where it binds and recruits phosphorylated ubiquitin. (3) As a result of Parkin-mediated ubiquitination, damaged and dysfunctional mitochondria are targeted for degradation by autophagosomes.

The loss of functional mitochondria in postsynaptic areas leads to an energy deficit, which blocks morphological plasticity. Ultimately, this process results in dendritic spine pruning and synapse loss (MacAskill et al., 2010; Dromard et al., 2021). Therefore, maintaining a “healthy” pool of mitochondria in the postsynaptic area is essential. However, MT are often damaged there due to increased functional load. The removal of defective mitochondria, which have accumulated improperly folded proteins and lost mitochondrial potency—through a process called mitophagy—is necessary to replace the damaged organelles with new ones (Figure 5). The PINK1/Parkin signaling pathway is primarily involved in the realization of mitophagy (Misgeld and Schwarz, 2017; Palikaras and Tavernarakis, 2020; Figure 5). When the serine/threonine-protein kinase PINK1 accumulates on OMM, it phosphorylates and activates Miro and GTPase protein Mfn2, as well as E3 ubiquitin ligase Parkin and ubiquitin itself. Activated Parkin binds to Miro or ubiquitin to attach to the OMM, where Parkin continues to bind and append phosphorylated ubiquitin, recruiting additional mitophagy adapters. As Parkin is ubiquitinated, the autophagosome degrades the damaged or dysfunctional mitochondria (Misgeld and Schwarz, 2017; Quinn et al., 2020). Disturbances in mitophagy processes may trigger synaptic failure, and cell death (Dagda et al., 2009; Makarov and Korkotian, 2023).

In summary, neuronal mitochondria, that are located away from the cell body, should work with higher level of self-sufficiency and sustain essential functional load. Furthermore, MTs are required to have instantaneous reaction due to the high-speed kinetic (milliseconds) of the major part of electrochemical events along dendritic branches and/or synaptic micro- compartments. The same way signaling pathways getting energetic requests should act. It is possible that mitochondrial dynamic relocation along calcium gradients and changes in the length and shape of mitochondrial clusters can be considered as possible solutions to the functional task. However, it is still unclear whether organelle mobility alone is sufficient to meet the complex energetic demands in neuronal and glial cells, or if more molecular tunings are required. In the next chapters, we will discuss which molecular mechanisms are potentially not only fast but also accurately direct ATP secretion in appropriate compartments.

## 4 Mitochondria in postsynaptic regions of central neurons

### 4.1 Mitochondria and local activity at postsynaptic sites

**FIGURE 6:**
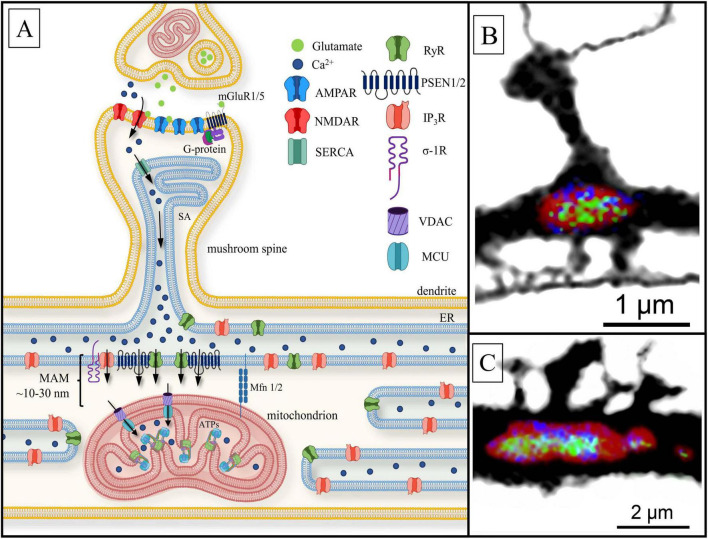
Postsynaptic Ca^2+^ signaling pathway to the mitochondria. **(A)** Black arrows present calcium currents. Ca^2+^ penetrates dendritic spine head via the activation of ionotropic glutamate receptors: N-methyl-d-aspartate receptor NMDAR and AMPAR. The sarcoplasmic/endoplasmic reticulum Ca^2+^-ATPase (SERCA) allows Ca^2+^ to enter the spine apparatus (SA). Ca^2+^ is released into the mitochondria-associated membranes (MAM) space through ryanodine-sensitive receptors (RyR), presenilin proteins (PSEN1/2) and inositol- 3-phosphate receptors (IP_3_R) located at the dendritic base on the endoplasmic reticulum (ER). PSEN1/2 and sigma-1-receptor (σ-1R) stabilize RyR and IP3R, respectively. Subsequently, calcium ions flux into the mitochondrial matrix through voltage-dependent anion channels (VDAC) and the mitochondrial calcium uniporter (MCU), located on OMM and IMM, respectively, where Ca^2+^ influences ATP synthase (ATPs) activity. Mitofusins (Mfn1/2) physically connects the organelles, keep free space between them with a length of 10–30 nm. Other abbreviations: mGluR1/5—the I and V type metabotropic glutamate receptor coupled with G-protein. **(B)** Confocal image of a single mitochondrion (MT) (red; MT morphology marker) located under dendritic spine base of a hippocampal dendrite, with immunolabeled ATP synthase (green) and MCU (blue); **(C)** the same as **(B)**. A cluster of dendritic spines with underlying MT. Note non-uniform distribution of MCU and ATP synthase along the MT surface (unpublished observation).

After the completion of the intense phase of synaptogenesis associated with neuronal development, dendritic mitochondria of mature neurons form spatially stable compartments about 30 μm in length (Rossi and Pekkurnaz, 2019; Palikaras and Tavernarakis, 2020; Pekkurnaz and Wang, 2022). Excitatory presynaptic terminals tend to contact dendritic spines, whereas most inhibitory inputs are localized on somatic and proximal dendritic areas (Leung and Peloquin, 2006; Jadi et al., 2012). Frequently, neighboring spines form spatial functional groups or clusters (see an example on Figure 6C; Nimchinsky et al., 2002; Dromard et al., 2021). Likewise, increased activity in such a clusters causes mitochondrial relocation toward presynaptic terminals (Lees et al., 2020; Seager et al., 2020), where energetically the mitochondria support the increased secretion of neurotransmitters, calcium maintenance, and presynaptic plasticity (Li et al., 2004; Chang et al., 2006; Obashi and Okabe, 2013). As mentioned earlier, the mitochondrial protein Miro functions as a Ca^2+^ sensor, inhibiting the connection between mitochondria and motor kinesin within the mitochondrial motor-adaptor complex. This leads to the undocking of the mobile fraction of mitochondria from microtubules and their docking in areas of local [Ca^2+^]_*c*_ spikes (Figure 4A; Sheng, 2014; Duarte et al., 2023). It is assumed that Ca^2+^-induced mitochondrial transport arrest near active synapses provides alternate energy to glutamatergic synapses (Colgan and Yasuda, 2014; Duarte et al., 2023).

The MT located under the spine bases with synchronized synaptic inputs are not only influenced by them but also exert influence on the structure of new dendritic spine groups themselves within ∼5 (Dromard et al., 2021) to 30 μm (Bapat et al., 2024) from the plasticity induction focus. The appearance of new spines around the potentiation focus is perhaps related to the fission and extension of the mitochondrial network (Dromard et al., 2021). Despite the evident significance, the connection between the local volume and morphology of mitochondria and synaptic functional activity remains apparently unclear. In the study by Thomas et al. (2023), it was discovered that the dendritic spine head volume and its postsynaptic density size do not correlate with the volume of adjacent MT. Instead, mitochondria are preferably concentrated around heterogeneous spine clusters receiving structurally and functionally diverse inputs in dendritic areas undergoing structural dynamics.

### 4.2 Mitochondria-driven modulation of synaptic plasticity

In the context of synaptic plasticity [long-term potentiation, (LTP) and/or long-term depression, (LTD)], free MT retain motility, allowing them to move along cytoskeleton elements, sometimes over quite long distances (Chang et al., 2006; Colgan and Yasuda, 2014). In postsynaptic compartments, MT do not only react to the current synaptic activity but are also able to affect synaptic currents, specifically by long-term regulation of synaptic strength. It is assumed that MT can exert a modulating effect on LTP/LTD in different ways (see chapter 6.2). For instance, with endoplasmic reticulum (ER) they form mitochondria-associated membranes (MAMs). These specialized regions emerge in narrow areas where the two structures take close positions, with a distance of approximately 10–30 nm (Li et al., 2004; Rangaraju et al., 2019a). MAM-contacts facilitate Ca^2+^ transfer from the ER to nearby MT, which maintains Ca^2+^ homeostasis in dendrites (Colgan and Yasuda, 2014; García-Bermúdez and Cuezva, 2016; Kushnireva and Korkotian, 2022; Kuijpers et al., 2024). MAM-contacts are regulated by Miro through clustering along OMM (Duarte et al., 2023). In addition, mitofusin 2 (Mfn2) acts as a MAM-contact protein and influences mitochondrial transport along microtubules by associating Miro (Johri and Chandra, 2021).

MT are able to buffer ions over physiological [Ca^2+^]_*c*_ range, within certain limits (around ∼100 μM; MacAskill et al., 2010) immediately in response to a local increase in [Ca^2+^]_*c*_ levels, thereby restricting Ca^2+^ spread along the dendrite in lateral directions (MacAskill et al., 2010; Kushnireva et al., 2022). Moreover, MT, having collected Ca^2+^, provide gradual, slow release of the ions to control the basal Ca^2+^ level and restrict functional interactions between neighboring dendritic spines (MacAskill et al., 2010). In the absence of synaptic transmission, [Ca^2+^]_*m*_ concentration is approximately equal to [Ca^2+^]_*c*_ concentration and is around 100 nM (Segal and Korkotian, 2014; Seager et al., 2020). Postsynaptic depolarization leads to a rapid increase in [Ca^2+^]_*c*_ (Gu et al., 1994; Pivovarova et al., 1999), and calcium is sequestered by calcium stores when [Ca^2+^]_*c*_ reaches 500 nM (Gu et al., 1994; Garbincius and Elrod, 2022). It has been established that changes in [Ca^2+^]_*m*_ follow local fluctuations in [Ca^2+^]_*c*_ with a slight delay not exceeding a few milliseconds (Kushnireva et al., 2022). Moreover, changes in [Ca^2+^]_*m*_ significantly correlate with fluctuations in [Ca^2+^]_*c*_, while the latter are framed by physiological conditions. Presumably, the mechanism ensures selective postsynaptic potentiation for neighboring spines at a distance around ∼2–5 μm (Kushnireva et al., 2022). Thus, if mitochondria-buffered cytosolic calcium has a direct or indirect relationship with mitochondrial activity in space, it is expected that Ca^2+^ is also be distributed unevenly in the cluster. Indeed, direct measurements indicate that [Ca^2+^]_*m*_ reactions are higher in area clusters under the spine base in comparison to more “lateral” clusters (Kushnireva et al., 2022). There is a high detection probability of [Ca^2+^]_*m*_ events under the spine base, compared to the probability in lateral zones of the same cluster (Kushnireva et al., 2022). Furthermore, it has been established that during mitochondrial depolarization related to functional activation, there is a short-term increase in reactive oxygen species (ROS) production by mitochondria (ΔΨ_mt_ on IMM ≈ –140 mV and lower) (Kaim and Dimroth, 1999; Lee et al., 2002). ROS could act as trigger to LTP induction (Thiels et al., 2000; Massaad and Klann, 2011) (see section 7).

In conclusion, mitochondria are directed to local Ca^2+^ bursts regions within dendrites to ensure the supply of ATP, proximal to dendritic spines. Dysfunctional mitochondria are eliminated through the mitophagy process to preserve dendritic spine functionality. However, a critical question remains: is there a distinct distribution of ATP synthase within IMM? Specifically, if mitochondria exhibit a non-uniform ATP synthase distribution, what impact does this have on ATP dynamics within the MT and on the surrounding cellular processes reliant on ATP?

## 5 Regulation of ATP synthesis

### 5.1 ATP as a glial signaling molecule

It is assumed that precise spatial organization and regulation of ATP synthase activity play significant roles both on presynaptic and postsynaptic sites. However, these roles remain largely unknown. One complicating factor is the fact that the synaptic region is engulfed by astrocytes and microglia which assume important roles in isolating the synapse. During heightened neuronal activity, astrocytes extensively release ATP to neurons while simultaneously increasing the amplitude and frequency of their calcium waves (see for review: Lezmy, 2023; Illes et al., 2025). This process is central to the concept of the tripartite synapse, where astrocytes not only support neurons but also regulate synaptic communication. ATP is released through pannexin and connexin channels and then converted into adenosine, modulating synaptic transmission (Dahl, 2015; Boué-Grabot and Pankratov, 2017; Illes et al., 2019). Additionally, astrocytes are involved in the glutamate shuttle, absorbing excess glutamate from the synaptic cleft and converting it into glutamine, which is then transported back to neurons for reuse (Rose et al., 2020; Zhang et al., 2023). This mechanism maintains excitatory signal balance and prevents neurotoxicity. Research indicates that astrocytic calcium waves can propagate to nearby cells, coordinating activity across neuronal networks (Fujii et al., 2017; Letellier and Goda, 2023). This highlights astrocytes as crucial modulators of synaptic plasticity and neuronal communication.

In addition to the effect on ion balance and neurotransmitter control, there is evidence of energy support of the most active synapses by astrocytes. They carry highly specialized structures, endfeet, through which glucose is absorbed from the cerebrovascular network. As hepatocytes, astrocytes are able to reserve glucose in the form of glycogen. It is believed that the mobilization of these reserves correlates with current neuronal activity. The targeted supply of synapses occurs through thin processes in the tripartite synapses mentioned above. For the implementation of this mechanism, the local process must be “aware of” the level of local synaptic activity. Moreover, the branched network of the astrocytic ER can serve as a carrier for intracellular glucose transport. However, it is not glucose but its derivative lactate that is released to the extrasynaptic space (Müller et al., 2018; Pellerin, 2018; Beard et al., 2022; Zhang et al., 2023). In this process, astrocytes metabolize glucose via glycolysis, producing lactate, which is transported to neurons via monocarboxylate transporters. Neuronal mitochondria then convert lactate to pyruvate, which serves as a substrate for ATP production (Beard et al., 2022; Pekkurnaz and Wang, 2022). However, the efficacy of astrocyte-neuron lactate shuttle may be affected by aging (Drulis-Fajdasz et al., 2018; Bonvento and Bolaños, 2021) because of glycogen metabolism enzymes concentration increasing in neurons’ MT (Drulis-Fajdasz et al., 2018).

In pathological conditions, astrocytes and microglia can shift from metabolic support to an inflammatory profile, altering their interactions with neurons. Instead of supplying ATP and maintaining neurotransmitter balance, they begin to release pro-inflammatory cytokines (IL-1β, TNF-α), which can impair neuronal function. Microglial purinergic P2X7 receptors become highly active in response to excess ATP, exacerbating inflammation and promoting neurotoxicity (see for review: Giovannoni and Quintana, 2020; López-Teros et al., 2024). Meanwhile, astrocytes may lose their ability to efficiently recycle glutamate, leading to excitotoxicity. This metabolic-inflammation switch contributes to the progression of neurodegenerative diseases, highlighting the dual role of glial cells in both support and dysfunction.

### 5.2 Regulation of local ATP synthesis in neuronal mitochondria

ATP in both presynaptic and postsynaptic MT is regulated by synaptic activity, and aberrant synaptic activity may lead to mitochondrial dysfunction. It has been established that under normal conditions action potentials (AP) stimulate local ATP production in axons (van Hameren et al., 2019). It is reasonable to assume that an analogous effect on ATP regulation may occur not only in the presynaptic terminal of an activated synapse but also in postsynaptic structures. Generally, because of increased [Ca^2+^]_*c*_, a number of local events take place: (1) Buffering of Ca^2+^ by MT increases [Ca^2+^]_*m*_, which regulates the entry of glutamate and pyruvate into the TCA cycle and enhances ATP production within the range of approximately –130 to –170 mV. This enhancement is due to a greater number of protons moving through the IMM voltage field per ATP synthase cycle, possibly as a consequence of adaptive stoichiometry or increased c-ring turnover (Wescott et al., 2019); (2) Intense mitochondrial fission takes place within 7–8 μm from the active dendritic spines as a result of NMDA-dependent LTP involving Ca^2+^ /calmodulin-dependent protein kinase II (CaMKII), Drp1 and actin polymerization (Divakaruni et al., 2018). Hyperpolarization enables Ca^2+^ uptake into the mitochondrial matrix, which leads to an increase in ATP production. This can occur during mitochondrial fusion (Klotzsch et al., 2015; Divakaruni et al., 2018). However, it is likely that ATP synthesis, increased after mitochondrial fission, is contributory to sustaining LTP, which is facilitated by intensified Ca^2+^ buffering because of new divided mitochondria is presented (Divakaruni et al., 2018).

There is just fragmentary evidence for ATP synthase distribution in neurons. Thus, it is known that ATP synthase competes in proton motive force with proteins such as UCP4 (uncoupling protein) concentrated on IMM as well (Klotzsch et al., 2015). UCP4 is included in the family of mitochondrial anion carrier proteins (MACP) (Smorodchenko et al., 2011). The notable feature of UCP4 relates to its expression in neuronal tissue (Ledesma et al., 2002; Crivelli et al., 2024). UCP4 utilizes oxidative phosphorylation energy similar to ATP synthase, however, not for ATP synthesis but for heat generation. Due to the proton gradient charge, the functioning of both proteins causes mitochondrial depolarization. We assume that their activity and expression could differ spatially as well as temporally. Spatial separation of ATP synthase and UCP4 is observed within MT and between the mitochondria of different sections of a neuron. ATP synthase is more expressed in cristae, while UCP4 is expressed in IMM regions adjacent to OMM (Klotzsch et al., 2015). Meanwhile, the proteins anchored in cristae are influenced by a higher ΔΨ_mt_ than the proteins adjoining the OMM. The maximal transmembrane proton gradient is limited by rapid lateral proton diffusion from cristae to UCP4 (Klotzsch et al., 2015). However, ATP synthase molecules are able to relocate from cristae to the inner boundary membrane (Weissert et al., 2021). Likewise, it has been observed that ATP synthase expression is mostly typical for dendritic branches, where local events requiring high energy leakage occur (Mironov, 2009), while UCP expression is pronounced in the neuron’s soma (Smorodchenko et al., 2009; Klotzsch et al., 2015). Both enzymes’ expressions are partly separated in time. Thus, ATP synthase is actively expressed in more mature, postnatal neurons, while UCP4 and ATPase as enzymes that hydrolyze ATP are more typical for mitochondria in embryonic cells when ΔΨ_mt_ does not reach –100 mV, thereby hindering ATP production by the synthase at this stage (Surin et al., 2013). ATP synthase molecules in mitochondria are typically concentrated at the cristae rims (Ježek et al., 2023). However, mitochondrial ATP synthases in all eukaryotic cells can form dimer or tetramer complexes across the crista surface, which are arranged in linear rows forming helical patterns (Acehan et al., 2011; Davies et al., 2011, 2012). The same can be said about neuronal mitochondria cristae (Nirody et al., 2020). The average distance between neighboring rows is around ∼28–30 nm, and the mean interval between neighboring dimer centers in a row is approximately ∼12–18 nm (Acehan et al., 2011; Davies et al., 2011). Dimerization and oligomerization also contribute to the high-degree curvature of cristae (Acehan et al., 2011; Domínguez-Zorita et al., 2022). The angle between the axis of rotation of ATP synthase V-shaped dimers in mammalian cells is dynamic, which permits the enzyme to function and/or contribute to the structural changes of cristae (Nirody et al., 2020; Domínguez-Zorita et al., 2022).

### 5.3 Ultrastructural and functional features of ATP synthase in dendrites

Neuronal mitochondrial ATP synthase localization, its distribution on cristae, its oligomerization, and structure are influenced by IF1. It has been established that the absence of IF1 reduces the quantity of neuronal mitochondrial cristae, making them shorter and wider (Romero-Carramiñana et al., 2023). IF1 also regulates ATP synthase activation in neuronal mitochondria, promoting the delicate adjustment of ATP production and the distribution of the ΔΨ_mt_ proton gradient within and between cristae (Campanella et al., 2009).

The conversion of ATPase from hydrolyzing to synthesizing ATP is accompanied by a shift in the F0-complex c-ring direction and a conformational alteration of the entire synthase (Surin et al., 2013), particularly the β-subunit of the F1-complex (Weber and Senior, 2003; Nath, 2023), (see Figure 3B). The conformation of ATP synthase can change due to the influx of Ca^2+^ into the mitochondrial matrix through MCU and the subsequent binding of ions to the β-subunit of ATP synthase (Hubbard and McHugh, 1996; Giorgio et al., 2017).

Although ATP synthase molecules are concentrated in mitochondrial cristae, a number of studies have described the ability of ATP synthase to move from MT to the plasma membrane of excitable cells (Xing et al., 2011; Chang et al., 2023). ATP synthase located in plasmatic membrane, called ectopic (eATPase) is capable of generating and secreting ATP into an extracellular environment to regulate extracellular ATP/ADP ratio and intracellular pH level (Xing et al., 2011). Before anchoring in the plasma membrane, the future eATPase complex is located in the mitochondria. Its transport to the mitochondria occurs as follows: kinesin family member 5B (KIF5B) interacts with Drp1 and binds to Miro and TRAK proteins, directing the bound complex along microtubules within mitochondrial fragments, or mitochondrial “vesicles,” to the plasma membrane (Chang et al., 2023). It has been established that both mitochondrial membranes can attach to the cell’s plasma membrane to anchor eATPase in it (Chang et al., 2023).

Nowadays, there is a consensus that the cytosolic calcium gradient precisely regulates ATP synthesis and/or secretion in eukaryotic cells (Boyman et al., 2020), including neuronal cells (Giorgio et al., 2017). This is especially relevant in the postsynaptic neuronal zone, where calcium signaling plays a key role in synaptic strength regulation. Additionally, there is a significant open question: how exactly is the local ATP level regulated, and is this regulation connected with the spatial distribution of ATP synthase in the postsynaptic zone? In other words, what signaling mechanisms provide the initiation of ATP synthesis and its secretion on a fast dynamic timescale, around milliseconds?

## 6 Calcium gradients in dendrites

### 6.1 Calcium signaling in mushroom spines

**FIGURE 7:**
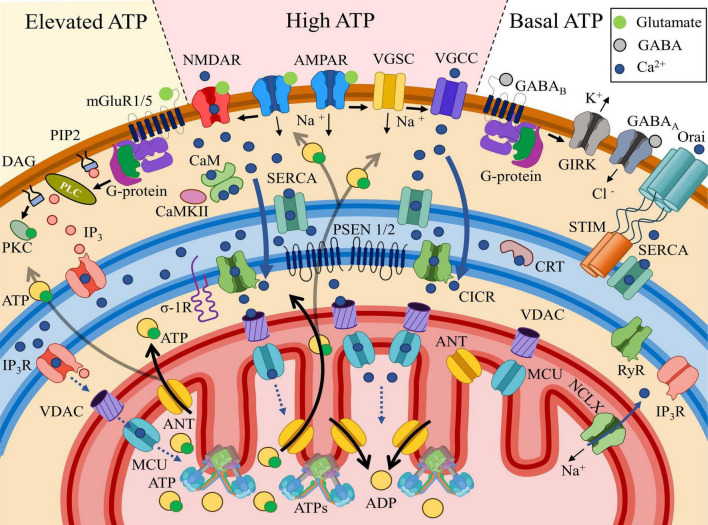
General trends of postsynaptic Ca^2+^ distribution and the associated ATP concentrations. **High ATP** section: AMPAR (α-amino-3-hydroxy-5-methyl-4-isoxazolepropionic acid receptors, placed in the middle at the top) activation by glutamate triggers plasma membrane depolarization. NMDAR (N-methyl-D-aspartate receptors, on the left in the center) requires both glutamate binding and membrane depolarization for activation. NMDAR efficiently conducts Ca^2+^, which is taken up by the SERCA pump and transported to the local compartment of the endoplasmic reticulum (blue field), where Ca^2+^ is buffered by calreticulin (CRT). Voltage-gated sodium channels (VGSCs) are activated due to membrane depolarization, which in turn opens voltage-gated calcium channels (VGCCs), allowing Ca^2+^ to leak from the intracellular space into the cytosol. Localized in the ER (endoplasmic reticulum), RyRs (ryanodine receptors) open due to interaction with elevated cytosolic Ca^2+^ via the calcium-induced calcium release (CICR) mechanism, leading to a high level of Ca^2+^ release within the MAM (mitochondria-associated membrane, space between the ER and the mitochondria) region. Ca^2+^ release is also facilitated by PSEN 1/2 modulating RyRs and/or serving as leak channels. From the MAM, Ca^2+^ penetrates through the OMM via VDAC (voltage-dependent anion channel) and MCU (mitochondrial calcium uniporter), which may facilitate ATP synthase (ATPs), leading to increased ATP production from ADP exchanged across the IMM via ANT (adenine nucleotide translocator). Activation of the mitochondrial Na^+^/Ca^2+^ exchanger (NCLX) facilitates Ca^2+^ efflux from MT into the cytosol, regulate MT Ca^2+^ homeostasis. **Elevated ATP** section: upon activation, the G-protein bound to type I and V metabotropic glutamate receptors (mGluR1/5) dissociates into subunits. The α-subunit of the G-protein activates phospholipase C (PLC), which cleaves phosphatidylinositol 4,5-bisphosphate (PIP_2_) into diacylglycerol (DAG), which remains membrane-bound, and inositol trisphosphate (IP_3_), which diffuses into the cytosol. DAG subsequently activates protein kinase C (PKC), initiating signaling cascades involved in long-term plasticity. IP_3_ binds to the inositol 1,4,5-trisphosphate receptor (IP3R), facilitating its opening and Ca^2+^ leakage into the cytosol. IP3Rs, oriented toward the MAM region, release Ca^2+^ toward mitochondria (MT) in lower amounts compared to the previously described pathway, contributing to a moderate ATP increase. **Basal ATP** section: The interaction of the inhibitory neurotransmitter GABA (gamma-aminobutyric acid) with ionotropic GABA_A_ (GABA type A) or metabotropic GABA_B_ (GABA type B) receptors conducts Cl^–^ (chloride) anions into the cytosol or removes K^+^ (potassium) via GIRK (G-protein-coupled inwardly rectifying potassium channels), thereby hyperpolarizing the membrane without creating an influx of Ca^2+^. As a result, cytosolic Ca^2+^ levels remain low. The only source of Ca^2+^ store replenishment in this case is the activation of Orai (voltage-independent calcium release-activated protein), which connects with oligomerized STIM (stromal interaction molecules) located near the plasma membrane and facilitates Ca^2+^ influx into the store/ER. The absence of increased Ca^2+^ and IP3 in the cytosol prevents Ca^2+^ release from the store, as RyR and IP3R remain closed. Under these conditions, ATP levels remain at the basal level.

As mentioned above, the AP-evoked Ca^2+^ influx into the synaptic area and dendritic cytosol occurs through NMDAR in response to glutamate release from the presynaptic terminal, which is triggered by postsynaptic activation of AMPAR (α-amino-3-hydroxy-5-methyl-4-isoxazolepropionic acid receptors) current (Jonas et al., 2004; Keller et al., 2008; Ashhad and Narayanan, 2013; Strobel et al., 2017). AMPAR and NMDAR are two major subtypes of glutamate receptors that play distinct roles in synaptic transmission and plasticity. AMPARs mediate fast excitatory synaptic transmission by quickly responding to glutamate binding, while NMDARs are voltage-dependent and require both glutamate and membrane depolarization to activate, leading to Ca^2+^ influx that influences synaptic modulation and plasticity (Figures 6A, 7).

Excitatory synapses are located on both dendritic spines and dendritic shafts. Shaft synapses are typical of developing neurons (Papa et al., 1995; Cottrell et al., 2000; Bucher et al., 2020). In more mature neurons, non-spine contact types are not able to form spatially bounded Ca^2+^ gradients due to the relative freedom of ion diffusion along the dendrite. However, why is the calcium gradient so crucial? The gradient is able to influence enzymatic chains, local protein synthesis, maintenance, and ATP production (Korkotian and Segal, 2006; Higley and Sabatini, 2012). In this context, it is essential to consider the factors influencing the uneven dynamic Ca^2+^ distribution in dendrites.

First, a synaptic Ca^2+^ gradient appears around the base of the dendritic spine. One of the forms of a mature dendritic protrusions is the mushroom-shaped spine, which consists of a head and a narrow neck connecting it to the dendritic shaft. However, there are other morphological types of dendritic spines, in particular thin spines that lack a distinct head and stubby spines, that are virtually neckless. The role and degree of functional maturity of these morphologies are still under debate (see for review Hering and Sheng, 2001). Briefly, thin spines, due to the lack of sufficient surface area, do not carry a significant receptor complex. In addition, branches of the ER rarely penetrate them. In contrast, stubby spines are too closely associated with the parent dendrite and do not actually represent an isolated chemical and electrical compartment. Hence, we will focus on mushroom spines.

Due to the synaptic current, [Ca^2+^]_*c*_ in the spine head may be significantly higher compared to the dendritic [Ca^2+^]_*c*_ (Holcman et al., 2005; Segal, 2010; Yuste, 2013). This deviation is achieved by the following factors: (1) the head volume is not large, approximately 1 × 10^–18^ – 10^–20^ L (Koch and Zador, 1993; Harris and Kater, 1994); (2) the head contains significant concentrations of Ca^2+^-binding proteins, such as CaMKII and calmodulin (Yuste et al., 2000); (3) free diffusion from the head through the neck, with a diameter of ∼0.1–0.2 μm (Arellano et al., 2007; Ofer et al., 2021), is hindered, creating an effect of ion compartmentalization (Arellano et al., 2007). Due to this accumulation of Ca^2+^ in the spine head, related to its buffering and diffusion obstruction from the neck, the transition process duration in the postsynaptic area increases (Hayashi and Majewska, 2005). Thus, Ca^2+^ gradient appearances around the spine neck base (Yuste et al., 2000; Korkotian and Segal, 2007). This is possibly one of the reasons that 90–95% of excitatory synapses are changed over to spines during the development of most brain structures (Nimchinsky et al., 2002; Bucher et al., 2020; Kandel, 2021).

In comparison with excitatory synapses, most inhibitory ones are located on proximal dendrites and the soma surface (Megías et al., 2001; Bucher et al., 2020), where they repress Ca^2+^- and Na^+^-currents responsible for synaptic plasticity and AP-evoked spikes, respectively, through membrane hyperpolarization (Miles et al., 1996; Megías et al., 2001). This location of inhibitory synapses relative to excitatory inputs is required for effective enhanced inhibition (Kandel, 2021). However, in several cases, inhibitory synapses are observed around dendritic spines (Müllner et al., 2015), or on the spines themselves. This occurrence is especially common for pyramidal neurons of neocortex (Marlin and Carter, 2014; Bucher et al., 2020) and CA1 hippocampal neurons (Huang et al., 2005). Inhibitory dendritic synapses are located on proximal dendrites (Chalifoux and Carter, 2011; Gidon and Segev, 2012; Ravasenga et al., 2022), as well as on apical ones (Chalifoux and Carter, 2011; Marlin and Carter, 2014). The main receptors involved in these synapses are the metabotropic gamma-aminobutyric acid B receptors (GABA_B_Rs) (Huang et al., 2005; Chalifoux and Carter, 2011). There is evidence that dendritic and spine GABA_B_Rs receptors are regulated by G-protein-coupled inwardly rectifying K^+^ (GIRK) channels, as well as NMDARs, which mediate Ca^2+^ influx into the postsynaptic area, leading to CaMKII activation (Huang et al., 2005; Schulz et al., 2021). It is possible that synaptic GABA_B_Rs play a key role in dendritic plasticity and excitability (Huang et al., 2005), by maintaining local Ca^2+^ homeostasis (Chalifoux and Carter, 2011; Figures 6A, 7).

Some forms of spines (for instance, stubby spines) do not contain a thin neck, which provides a Ca^2+^ gradient. In the case of mushroom-like structures, the neck functions as a link rather than a barrier for Ca^2+^ diffusion into the dendrite (Korkotian and Segal, 2000). Diffusion kinetics of Ca^2+^ through the neck are also influenced by ER tubules (Segal and Korkotian, 2016). In the case of complete separation from the rest of the reticulum, the ER assumes an isolated structure in the form of the spine apparatus (SA), which possesses an actin-associated complex based on proteins such as synaptopodin. Local ER (Sheng and Cai, 2012) and SA (Vlachos et al., 2009; Segal, 2010) control [Ca^2+^]_*c*_ levels in response to ion influx from the outside. Ca^2+^ received through NMDAR into the spine head is accumulated into the local ER storage, while being secreted through ryanodine receptor (RyR) or inositol trisphosphate receptor (IP_3_R) anchored on ER into dendrite (Kushnireva and Korkotian, 2022; Figure 6).

Ca^2+^ released from ER acts as a messenger, particularly in MAM-contacts (Johri and Chandra, 2021; Figure 6). There are major proteins related to MAM: (1) RyRs, located on ER/SA under the spine base and facing the dendritic shaft (Basnayake et al., 2021, 2022); (2) presenilins (PSEN1/2) (Korkotian et al., 2019) acted as RyR regulators (Rybalchenko et al., 2008) and (3) sigma-1 receptors (σ1Rs) that regulate and stabilize IP_3_R-MAM contacts (Hayashi and Su, 2007; Raturi and Simmen, 2013; Johri and Chandra, 2021). It is assumed that PSENs influence Ca^2+^-leakage from ER in two ways. Firstly, PSEN1/2 modulates RyR conductance by interacting with the receptor’s cytosolic domain at the N-terminus (Rybalchenko et al., 2008; Payne et al., 2015). In this regard, there is experimental evidence that RyR levels are increased in the presence of mutant PSENs (Lerdkrai and Garaschuk, 2018), possibly serving as a compensatory mechanism for PSEN1 regulation deficit (Kushnireva and Korkotian, 2022; Makarov et al., 2023). Secondly, there is evidence that PSEN1 can function as an ion leak channel through the large hydrophilic loop located on the cytosolic side (Tu et al., 2006; Bagaria et al., 2022). MAM-contact proteins also include resident ER proteins such as calnexin or calbindin, the chaperone protein glucose-regulated protein 75 and deglycase, which together with IP_3_R and VDAC regulate the transfer of Ca^2+^ from the ER to the mitochondrial matrix through MCU (Johri and Chandra, 2021; Markovinovic et al., 2022). Additionally, the role of mitofusins (Mfn1/2) in the organization of MAM-contacts should be noted (Barazzuol et al., 2021; Markovinovic et al., 2022). Mfn1/2 is involved in anchoring mitochondria under the active spine base: the proteins physically hold ER/SA and the mitochondria at quite a close distance, ∼10–30 nm (Markovinovic et al., 2022). MAM-contacts are involved in Ca^2+^ level maintenance, as well as in the regulation of apoptosis, mitochondrial dynamics, mobility, and autophagy (Vance, 2014; Johri and Chandra, 2021; Zhao and Sheng, 2024). The alteration of distances within MAM by Mfn1/2 can affect the rate and efficiency of Ca^2+^ buffering, which in turn influences mitochondrial energy metabolism and signaling pathway regulation. Ca^2+^ released by RyR, PSEN1/2, and/or IP_3_R enters the mitochondrial matrix through VDAC and MCU, located just under MAM-contacts on the OMM and IMM, respectively (Garbincius and Elrod, 2022; Markovinovic et al., 2022; Kuijpers et al., 2024). Below, we will discuss how this mechanism is able to provide directed ATP secretion. Dendritic spine(-s) with MT are imaged on the confocal microscope in Figures 6B,C, respectively. Panel B represents a single mushroom spine in contact with an axon. Panel C contains a cluster of spines. MT (single on B and clastered on C) are seen at the base of both examples (red) along with ATP synthase (green) and MCU (blue). Possibly the larger spine on C is assocoated with higher concentrations of ATP synthase and MCU.

### 6.2 Regulation of ATP levels by ongoing synaptic activity

In Figure 7, the general trends in postsynaptic Ca^2+^ distribution and the associated ATP concentrations are illustrated. In the excitatory synapses, AMPARs depolarize the membrane after binding to the excitatory transmitter, presynaptic glutamate which allows NMDAR activation, dependent on both ligand binding and membrane potential. NMDAR efficiently conducts Ca^2+^, which can be taken up by the sarcoplasmic/endoplasmic reticulum Ca^2+^-ATPase (SERCA) pump and moved to the local compartment of the ER or to the stand-alone SA. In addition, sufficient depolarization of the plasma membrane transmits excitation to the voltage-gated sodium channels (VGSC). The entry of Na^+^ ions involves the voltage-gated calcium channels (VGCC). The following influx of Ca^2+^ is also absorbed into the depot and calreticulin (CRT) internal buffer protein. MAM-localized RyRs open due to interaction with elevated cytosolic Ca^2+^ via the calcium-induced calcium release (CICR) mechanism, after which calcium is released towards MT. This is also facilitated by PSEN1/2 modulating RyRs and/or serving as leak channels, as well as by the σ-1 receptor. From the MAM, where [Ca^2+^]_*c*_ can reach 20–40 μM (Hajnóczky et al., 2000), Ca^2+^ penetrates through the OMM via VDAC and into and through the IMM via the MCU uniporter. In the internal environment of the MT, Ca^2+^ may directly facilitate the Krebs cycle and ATP synthesis, leading to increased ATP production from ADP, which is transported across the IMM via ANT. The released ATP diffuses into the postsynaptic zone, where its increased concentration (“High ATP”) is formed, for example, to maintain short-term plasticity/long-term plasticity mechanisms associated with the buffer protein calmodulin (CaM) and Ca^2+^/calmodulin-dependent protein kinase II (CaMKII). Following heavy Ca^2+^ influx into MT Na^+^/Ca^2+^ exchanger plays a key role in Ca^2+^ extrusion (Rodrigues et al., 2022), but the mechanism depends on Na^+^ cytosolic and mitochondrial concentration (Boyman et al., 2013).

Metabotropic receptors, which are coupled to a G-protein, for instance, mGluR 1/5 can activate phospholipase C (PLC). Activated PLC cleaves the phospholipid phosphatidylinositol 4,5-bisphosphate (PIP_2_) into lipophilic diacylglycerol (DAG) and hydrophilic IP_3_. DAG can interact with protein kinase C (PKC), triggering long-term plasticity chains. IP_3_ interacts with IP_3_R, another calcium depot receptor that creates a local calcium release. This released Ca^2+^ enters MT in significantly smaller quantities via the pathway described above, creating a zone of moderate ATP increase in the cytosol (“Elevated ATP”) (see Table 2 for half-activation constants).

**TABLE 2:** Half-activation Ca^2+^ concentration of calcium regulatory proteins.

Receptor type	Half-activation Ca^2+^ concentration*	Source
SERCA	≤0.4 μM	Pancreatic β-cells (Fridlyand et al., 2003)
RyR	Activated by low concentrations (1–10 μM)	Cardyomyocites (Laver and Curtis, 1996; Laver, 2007; Seidlmayer et al., 2016)
VDAC	Ca^2+^ permeabolity in open state 20 ions/s (1 μM), in closed state 80 ions/s (4 μM)	Rat liver (Tan and Colombini, 2007)
MCU	10 μM	Isolated MT (Graier et al., 2007)
IP_3_R	150 nM	Cardyomyocites (Ju et al., 2012) Brain cells (Verkhratsky, 2005)
NCLX	20 — 40 μM	Bacteria (Besserer et al., 2012)
IF1+CaM	5 μM	Cardiac myocytes (Saucerman and Bers, 2012)
DRP1 + Calcineurin	1 μM	Brain cells (Klee et al., 1979) DRP1 dependent on calcineurin (Lai et al., 2023)

*All values are mean or approximate.

Finally, the interaction of the inhibitory transmitter GABA with the ionotropic GABA_*A*_ or metabotropic GABA_*B*_ receptors conducts Cl^–^ anions into the cytosol or removes K^+^ from it via GIRK, thereby hyperpolarizing the membrane without creating an influx of Ca^2+^. The only source of depot replenishment in this case remains the store-operated calcium entry (SOCE) mechanism via the plasma channel Orai and the associated depot Ca^2+^ sensor STIM. In this case, Ca^2+^ is immediately pumped into the depot by the SERCA pump located nearby, without entering the cytosol (see Basnayake et al., 2022). The absence of an increased Ca^2+^ and IP_3_ in the cytosol does not cause ion release from the depot, since RyR and IP_3_R remain closed. In this case, the ATP level remains at the basal level (“Basal ATP”).

The relationship between local postsynaptic Ca^2+^ concentrations and ATP production or delivery remains unclear, as no precise data is available in literature. Fink et al. (2017) reported that in skeletal muscle, Ca^2+^ levels above basal (up to 450 nM) effectively enhanced mitochondrial respiration. With further increases, ATP production slowed down, and after 10 μM, it began to decline. Similar or close values were obtained in mathematical modeling of mitochondrial respiration (Tarraf et al., 2021). In addition, Kushnireva et al. (2022) showed that mitochondrial clusters in the postsynaptic zone effectively regulate local Ca^2+^ levels and strictly limit their spreading along the dendritic axis to 3–5 μm. In this context, tight local control over Ca^2+^ not only modulates ATP production but also finely tunes mitochondrial dynamics.

Thus, the emergence of significant calcium gradients extending beyond the postsynaptic compartment is possible only with massive synaptic potentiation. Under resting conditions, [Ca^2+^]_*c*_ levels in dendrites typically remain low (approximately 50–100 nM; Verma et al., 2022), and although backpropagating action potentials (bAPs) can evoke global calcium transients reaching 30–90 μM (Sterratt et al., 2012), these supraphysiological concentrations do not appear to affect mitochondrial mobility (Silva et al., 2021). In sharp contrast, local synaptic activation produces modest yet highly localized Ca^2+^ elevations often in the range from 100 to 200 nM (Gunter and Sheu, 2009) to only up to 10 μm inside dendritic spines during activation (Verma et al., 2022) which are sufficient to reduces the movement of dendritic MT and leads to the grouping of them at the base of the spines and even entry into them (Seager et al., 2020) by the EF-hand domains of the MT adaptor protein Miro, leading to uncoupling of motor proteins and promoting MT arrest near active synapses. Meanwhile, higher concentrations of Ca^2+^ can further disrupt MT-microtubule interactions. Thus, a concentration of 50 μM free Ca^2+^ has been shown to cause a 50% reduction in MT binding to microtubules (Wang and Schwarz, 2009). Consequently, mitochondria undock from microtubules and move directly to local Ca^2+^ spike areas (Figure 4A; Sheng, 2014; Alberti et al., 2022; Zaninello and Bean, 2023), triggered, for example, by NMDAR activity (Duarte et al., 2023; Kuijpers et al., 2024).

Moreover, pathological conditions associated with Ca^2+^ dysregulation can profoundly disturb mitochondrial transport. In particular, mutations in presenilin-encoding genes—which underlie many familial Alzheimer’s disease cases—result in aberrant Ca^2+^ release from ER, initiating their physiological imbalance (Sarasija et al., 2018)

These dysfunctional dynamics are believed to disrupt synaptic homeostasis and have been linked not only to Alzheimer’s disease but also to other neurodegenerative conditions such as Parkinson’s disease and amyotrophic lateral sclerosis (Sheng and Cai, 2012; Zaninello and Bean, 2023), in which imbalances in Ca^2+^ homeostasis exacerbate synaptic dysfunction and neuronal loss.

Thus, while modest local Ca^2+^ elevations (in the range of 100–200 nM) appear to fine-tune mitochondrial positioning for optimal ATP delivery at synaptic sites, excessive Ca^2+^—whether due to intense synaptic activity or pathological mutations—can instead derail mitochondrial trafficking, thereby contributing to neurodegenerative disease cascades.

In fact, mitochondria are not the only source of ATP in neurons, including their postsynaptic microdomains. It is well known that aerobic glycolysis can serve as an important, albeit secondary, energy pathway, especially in the soma and areas of increased metabolism. Glycolysis is a metabolic pathway that oxidizes glucose to pyruvate, generating ATP and NADH. Although glycolysis does not appear to be directly dependent on calcium concentration, this ion may act as an indicator of increased synaptic load (Díaz-García et al., 2021). Ca^2+^ may act as a trigger for increased glycolysis by activating pathways such as the Aralar/malate-aspartate shuttle, indirectly affecting mitochondrial function. In addition, increased intracellular calcium may act as a signal for activation of the glycolytic pathway. Thus, some G-protein coupled metabotropic receptors can activate PKC protein kinase via PLC-DAG-IP_3_ pathway with following release of Ca^2+^ from stores (Figure 7). In addition, some PKC isoforms are Ca^2+^-dependent. It has been established that in astrocytes, PKC regulates glycolysis by phosphorylation of the glycolytic enzyme pyruvate kinase (Horvat et al., 2021).

## 7 Calcium signaling within the mitochondria

### 7.1 Regulation of mitochondrial membrane potential by calcium ions

Ca^2+^ can permeate the mitochondrial matrix via the MCU, driven by its steep electrochemical gradient, and is regulated by the significant electric potential across the IMM (ΔΨ_mt_ ∼−150 mV) (Gu et al., 1994; Walters and Usachev, 2023). Early studies reported that during physiological [Ca^2+^]_*c*_ signaling, [Ca^2+^]_*m*_ uptake modulates the amplitude and duration of Ca^2+^ signals without significantly affecting ΔΨ_mt_ (Chalmers and McCarron, 2008). Depolarization of ΔΨ_mt_ is expected only if [Ca^2+^]_*c*_ fluctuations are repetitive and sustained: during AP and backpropagation AP (bAP) (Stoler et al., 2022); no effect is anticipated during single [Ca^2+^]_*c*_ transients triggered by calcium influx or release from intracellular stores (Chalmers and McCarron, 2008). However, the occurrence of transient depolarizations of ΔΨ_mt_ during cellular Ca^2+^ signaling may significantly depend on the cell type. For instance, in the study by Huang et al. (2004), distinct types of neurons and other cell types exhibited variations in ΔΨ_mt_: the mean ΔΨ_mt_ of fibroblasts was approximately −112 ± 2 mV, while that of neuroblastoma SH-SY5Y cells was around −87 ± 2 mV. The mean ΔΨ_mt_ within different neuronal structures also varied: in primary cultured neurons, it ranged from −111 to −78 mV in cell somata and from −113 to −84 mV in axonal growth cones. Similarly, the mean ΔΨ_mt_ of differentiated PC12 line cells ranged from −121 to −89 mV in cell bodies and from −130 to −69 mV in growth cones (Huang et al., 2004). However, in neurons, ΔΨ_mt_ in general (Dayal et al., 2024) and specifically dendritic (Zheng et al., 2013) ΔΨ_mt_, can reach higher values than those observed in the aforementioned cell types (∼−150 to −180 mV). Moreover, it has been shown that axonal MT exhibit significantly higher sensitivity to Ca^2+^ for the activation of mitochondrial Ca^2+^ uptake compared to non-neuronal mitochondria (Ashrafi et al., 2020). A recent study demonstrated that APs induced by a series of synaptic stimuli elicit stronger [Ca^2+^]m responses in dendrites compared to those triggered by a series of current pulse injections (Stoler et al., 2022).

Detection of coincident synaptic inputs and enhanced mitochondrial Ca^2+^ uptake accelerates the rates of NADH production and consumption through the TCA cycle (see details below) and the electron transport chain, respectively, increasing ATP synthesis (Stoler et al., 2022). This positions mitochondria as finely tuned contributors to the processes of synaptic plasticity in neurons. Research on neurons shows that mitochondrial calcium uptake can be triggered by an increase in [Ca^2+^]_*c*_ as small as 200–300 nM above basal levels ([Ca^2+^]_c_ ≈ 50–100 nM; [Ca^2+^]_m_ ≈ 100–200 nM at rest) (Gu et al., 1994; Pivovarova et al., 2002; Stanika et al., 2012; Hu et al., 2018; Walters and Usachev, 2023; Murphy and Eisner, 2024). The rate of Ca^2+^ efflux from MT is significantly lower than the rate of Ca^2+^ uptake (∼23 μM/s in neurons). However, [Ca^2+^]_*m*_ typically does not exceed 1–5 μM in neurons, even after intense stimulation, due to the ability of incoming calcium to be buffered in the mitochondrial matrix by forming phosphate complexes (Walters and Usachev, 2023). Thus, MT Ca^2+^ transients typically lag and persist longer than cytosolic calcium ([Ca^2+^]_*c*_) spikes, whether induced or spontaneous (Lin et al., 2019; Kushnireva et al., 2022; Stoler et al., 2022). It has been suggested that the prolonged release of buffered calcium from MT may influence Ca^2+^ signaling during recovery periods following stimulation or spontaneous activity, effectively preserving a “historical record”of prior activity (Gellerich et al., 2010; Pivovarova and Andrews, 2010).

### 7.2 Calcium regulation of metabolic cycles

**FIGURE 8:**
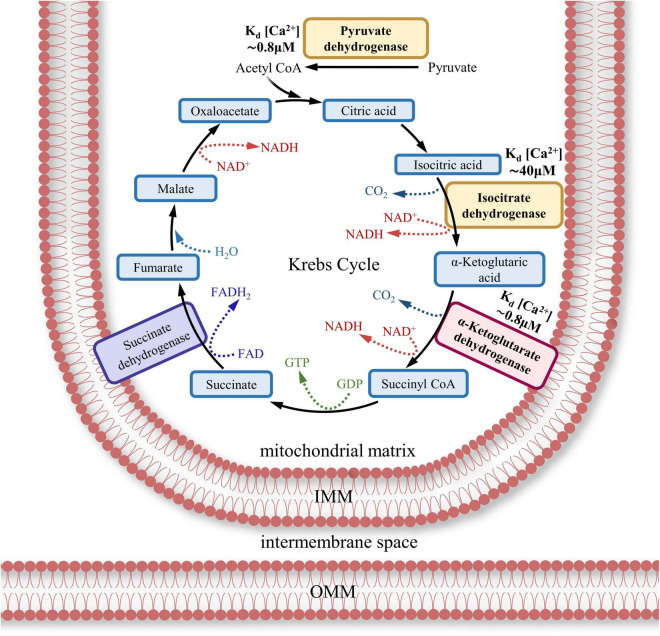
Ca^2+^-dependent dehydrogenases of the Krebs cycle (TCA, tricarboxylic acid cycle). Three Ca^2+^-dependent dehydrogenases (bold font) of Krebs cycle are represented in the scheme. Half-activation concentration of Ca^2+^ for pyruvate dehydrogenase and α-ketoglutarate dehydrogenase is approximately 0.8 μM and around 40 μM for isocitrate dehydrogenase (mentioned as Kd). α-ketoglutarate dehydrogenase is included in I complex of ETC.

Fluxing into the mitochondrial matrix, Ca^2+^ can influence ATP synthase activity through two mechanisms: indirectly, via the Krebs cycle, and directly, by binding to the β-subunit of the ATP synthase F1 complex. Concerning Ca^2+^’s influence on the TCA/Krebs cycle, it is important to note that some of the dehydrogenases involved in the TCA cycle are Ca^2+^-dependent (Figure 8). For example, pyruvate dehydrogenase and α-ketoglutarate dehydrogenase exhibit Ca^2+^ sensitivity, with a Ca^2+^ half-activation concentration of approximately 0.8 μM (Walters and Usachev, 2023). Isocitrate dehydrogenase is also Ca^2+^-dependent; however, its Ca^2+^ half-activation concentration is significantly higher, at around 40 μM (Walters and Usachev, 2023). Elevated Ca^2+^ concentrations within the mitochondrial matrix stimulate the enzymatic activity of these dehydrogenases, leading to increased flux through the TCA cycle. During a single Krebs cycle, two molecules of CO_2_, three molecules of NADH, one molecule of FADH_2_, and one molecule of GTP are produced. Electrons supplied by NADH and FADH_2_ are both critical for driving the respiratory chain, which encompasses the key stages of oxidative phosphorylation responsible for ATP production. Enhanced NADH generation increases the supply of electrons to the respiratory chain, further supporting ATP synthesis during heightened neuronal activity.

The direct influence of Ca^2+^ on ATP synthase function and dynamics remains a subject of debate (Gellerich et al., 2010). It has been suggested that the ion binds to the β-subunit of the ATP synthase F1 complex directly (Hubbard and McHugh, 1996), inducing conformational changes in the entire ATP synthase (Territo et al., 2000; Nesci, 2022). An increase in [Ca^2+^]_*m*_ caused by Ca^2+^ influx through the MCU leads to enhanced proton intake by accelerating the c-ring rotation. However, to date, there is no conclusive evidence for such direct interaction (Territo et al., 2000; Nesci et al., 2016; Wescott et al., 2019).

The highest activity of ATP synthase in eukaryotic cells is achieved when the K_*m*_ for Ca^2+^ ranges from ∼200 nM (Territo et al., 2000) to ∼600 nM in the presence of ADP (K_*m*_ ≈ 20 μM) (Wescott et al., 2019). Thus, it is arguable that the key condition for maintaining energetic balance in neuronal synaptic zones is the optimal concentration of [Ca^2+^]_*m*_ and the efficient import of ADP from the cytosol into the mitochondrial matrix, coupled with the rapid export of ATP from the mitochondrial matrix into the cytosol (Klingenberg, 2008; Kawamata et al., 2010). The effect of Ca^2+^ might be attributed to the direct activation of the most widespread member of the IMM protein transporter family involved in metabolite exchange, Adenine nucleotide translocator (ANT), also known as the ADP/ATP carrier (AAC) (Territo et al., 2000; Kunji et al., 2016; Wescott et al., 2019). ANT utilizes the energy stored in ΔΨ_*mt*_ to exchange ADP for ATP through an antiport mechanism (Wescott et al., 2019). Its submolecular structure consists of three homologous domains. Each domain contains two transmembrane α-helices connected by a helical bridge. It is hypothesized that the transporter performs a cyclic half-turn in the horizontal plane, parallel to the IMM, in two opposite directions. During the first half-turn, captured ADP is transferred into the mitochondrial matrix, and during the next half-turn, the synthesized ATP is exported from the matrix to the cytosol. In other words, the transporter alternates between two states: one opens to the matrix and the other opens to the intermembrane space (Kunji et al., 2016; Ruprecht et al., 2019). Thus, ANT exports mitochondrial ATP through IMM in exchange for cytosolic ADP at a 1:1 ratio (Pfaff and Klingenberg, 1968; Klingenberg, 1980).

In brain tissue two ANT isoform are expressed: ANT1 is maintained at a high-expression level (Mentel et al., 2021; Mishra et al., 2023), while ANT4 has comparatively the low-expression one (Mishra et al., 2023). Except for ANT, phosphate carrier protein is also involved in ATP transport. It catalyzes phosphate ion (P_*i*_) transport into mitochondrial matrix as a symporter (in collaboration with the proton) or as antiporter in exchange for hydroxyl ion. Without free access of nonorganic phosphate ATP synthesis is not possible. For another thing, there is evidence to suggest that P_i_ influences Ca^2+^ level in the mitochondria. P_i_ can not only stimulate Ca^2+^ influx (Zoccarato and Nicholls, 1982) by MCU but inhibit Ca^2+^ efflux to enhance [Ca^2+^]_*m*_ (Seifert et al., 2015). Another significant transporter is ATP-Mg^2+^/P_*i*_ carrier (short calcium-binding mitochondrial carriers (SCaMC), that is also Ca^2+^-dependent transporter having EF-hand structure in its compound to bind the Ca^2+^ (Mentel et al., 2021; Mishra et al., 2023). Thus, except for ADP and P_*i*_ availability in mitochondrial matrix, Ca^2+^ affects ATP synthesis (Wescott et al., 2019).

The function of ANT can be modulated by cardiolipin, a lipid also detected in neurons (Bradshaw et al., 2024). It is proposed that cardiolipin binds ANT monomers within a dimeric structure, thereby regulating ANT’s role as an ATP/ADP carrier (Paradies et al., 2014). However, this hypothesis remains unsupported (Paradies et al., 2009), as several studies describe cardiolipin as a Ca^2+^-dependent molecule, whose interaction with ANT may be unstable under certain conditions (Hwang et al., 2014; Miranda et al., 2020). Evidence also suggests that ANT is a Ca^2+^-sensitive carrier, and its function may be influenced by mitochondrial proteins such as cyclophilin D (CyD) (Calì et al., 2012; Bernardi et al., 2022).

### 7.3 Mitochondrial permeability transition pore

**FIGURE 9:**
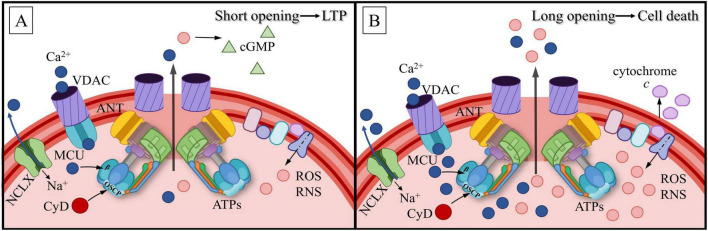
Mitochondrial permeability transition pore (mPTP) structure in physiolodical **(A)** and pathological **(B)** conditions. The mPTP is formed by voltage-dependent anion channels (VDAC) on the outer mitochondrial membrane (OMM) and adenine nucleotide translocator (ANT) together with the ATP synthase c-ring on the inner mitochondrial membrane (IMM). Ca^2+^ enters the mitochondrial matrix via VDAC and the mitochondrial calcium uniporter (MCU). Once inside, Ca^2+^ with cyclophilin D (CyD) modulate the permeability of ANT and ATP synthase. Na^+^/Ca^2+^ exchanger (NCLX) plays a key role in Ca^2+^ maintenance within mitochondrial matrix. **(A)** Physiological condition where short openings of the mPTP allow a low-level leak of reactive oxygen species (ROS) and reactive nitrogen species (RNS). This modest release activates cyclic guanosine monophosphate (cGMP) signaling, which contributes to long-term potentiation (LTP). **(B)** Pathological condition associated with prolonged mPTP opening and high [Ca^2+^]_m_ levels. In this state, a substantial leak of ROS and RNS occurs, leading to cytochrome c release and cell death.

Oxidation of cardiolipin affected by Ca^2+^ can impair ANT’s ATP/ADP exchange function, potentially triggering the opening of the mitochondrial permeability transition pore (mPTP) (Paradies et al., 2011, 2014; Miranda et al., 2020; Bradshaw et al., 2024). The opening of mPTP alters the permeability of the mitochondrial membrane (Karch et al., 2019; Figure 9).

mPTP opening results in the leakage of Ca^2+^ from mitochondria (Kinnally et al., 2011; Kaufman and Malhotra, 2014; De Marchi et al., 2014). The role of mPTP remains controversial: while calcium release under physiological conditions primarily occurs via the Na^+^/Ca^2+^ exchanger, mPTP is thought to play a crucial role in maintaining physiological levels of [Ca^2+^]_m_, without necessarily leading to mitochondrial dysfunction. Many studies link mPTP opening to mitochondrial damage and cell death, suggesting that mPTP activation occurs when [Ca^2+^]_m_ approaches toxic levels. This can cause mitochondrial depolarization and a reduction in ATP production. However, other research highlights the reversibility of [Ca^2+^]_m_ accumulation and mitochondrial swelling (Pivovarova and Andrews, 2010). Some researchers suggest that mPTP may consist of several components, including ANT (Mnatsakanyan and Jonas, 2020b; Bradshaw et al., 2024), the c-ring of ATP synthase dimers (Bernardi et al., 2022), and VDAC (Mnatsakanyan and Jonas, 2020b). Others propose that mPTP primarily comprises ANT and ATP synthase monomers (Bernardi et al., 2022). The following molecular chain has been proposed: CyD binds to OSCP (Mnatsakanyan and Jonas, 2020a), which accelerates the dissociation of ATP synthase dimers (Bernardi et al., 2022) and contributes to the structural disintegration of ATP synthase (Murphy, 2022; Endlicher et al., 2023). These changes facilitate the formation of mPTP.

The physiological effects of mPTP are as follows: Ca^2+^ entering the mitochondrial matrix triggers transient mPTP opening, leading to short-term IMM depolarization (Mnatsakanyan and Jonas, 2020a) and cristae remodeling (Mnatsakanyan and Jonas, 2020b; Bonora et al., 2022). The specific mechanisms underlying depolarization remain unclear. In the open state, the pore diameter is approximately 2–4 nm (Novgorodov and Gudz, 1996; Mironova and Pavlov, 2021). Prolonged mPTP opening, however, results in mitochondrial potential dissipation, ionic imbalance, and ROS generation (Lippe et al., 2019; Mnatsakanyan and Jonas, 2020a). Moreover, the ROS and reactive nitrogen species (RNS) produced in this context do not only contribute to oxidative stress; when generated at sub-toxic levels, as observed beneath the base of dendritic spines or near spineless synapses on the dendritic shaft, they may diffuse across the plasma membrane and stimulate guanylate cyclase or cGMP-dependent protein kinase. This signaling feedback can modulate neurotransmitter release and postsynaptic potentiation (Hidalgo et al., 2007; Massaad and Klann, 2011; Beckhauser et al., 2016), with physiologically relevant [H_2_O_2_] typically exceeding 1 μM (Hidalgo et al., 2007). It is hypothesized that mPTP’s physiological role is maintained as long as [Ca^2+^]m does not exceed 10–40 μM, beyond which ATP production is inhibited (Fink et al., 2017). In this state, the Krebs cycle dehydrogenases are activated. The molecular cascade involves Ca^2+^ binding to β-subunits, inducing conformational changes in the ATP synthase stalks and the c-ring of the F_0_ complex (Mnatsakanyan and Jonas, 2020a). Prolonged conformational alterations in the c-ring transform it into a nonselective channel, ultimately leading to dissociation of the ATP synthase F_1_-F_0_ complex, OMM disruption, and cytochrome c release (Mnatsakanyan and Jonas, 2020a).

As mentioned, the mitochondrial oxidative phosphorylation (OXPHOS) system consists of an electron transport chain and ATPs. In addition to ATP synthesis, OXPHOS generates ROS. This occurs due to proton leakage with the formation of a superoxide radical as a by-product, which is then transformed into H_2_O_2_. It is known that ROS act as an important second messengers, but when OXPHOS function is disrupted, ROS hyperproduction takes on dangerous forms, leading to oxidative stress. Among the OXPHOS regulators, IF1 attracts special attention. Its activity is manifested in MT damage and energy metabolism imbalance. In neurons IF1 acts by inhibiting the hydrolase and synthetic activity of ATPase, shifting energy metabolism towards glycolysis (Formentini et al., 2014). It may be assumed that such shift may occur during local energy deficiency, associated with elevated synaptic activity or with adjacent mitochondrial dysfunctions. The role of IF1 in ROS overload is still debated (Gore et al., 2022).

It is possible that mPTP formation can lead to wide spectrum of pathologies development to ATP synthase dysfunction initiating a number of neurological disorders, such as Alzheimer’s disease, Parkinson’s disease (Coluccino et al., 2023; Baev et al., 2024), amyotrophic lateral sclerosis, and more rarely Huntington’s disease, Wilson’s disease and Friedreich’s ataxia (Galber et al., 2021). In all the listed diseases, there is a disruption in ATP synthesis, which can lead to the death of energy-dependent neurons (Calì et al., 2012). There is evidence to suggest that OSCP synthesis is reduced in young and old brains in some animal models of Alzheimer’s disease as well as in old brains without any disorders (Mnatsakanyan and Jonas, 2020b). The cellular mechanisms that inhibit Ca^2+^ binding to the ATP synthase β-subunit are demonstrated during the progress of the early stages of neurodegeneration (Calì et al., 2012; Pchitskaya et al., 2018; Mnatsakanyan and Jonas, 2020a; Verma et al., 2022).

## 8 Conclusion

Generalization and analysis of a large body of data related to mitochondrial mechanisms in neurons allow us to make several preliminary conclusions with respect to the high kinetics of energy-intensive processes in synaptic zones associated with neurotransmitter secretion and postsynaptic mechanisms, including the receptor’s balance, its modulation and plasticity. It is remarkable that all the events occur with high speeds in the millisecond range and within an extremely limited volume of a fraction of μm^3^. These factors make calcium ions an ideal agent in signaling transmission, accounting for their high mobility and the difficulty of their regulation by intracellular storage compartments, including the SA (probably a local Ca^2+^-store in the necks of dendritic spines) and thin ER branches. Furthermore, cytosolic calcium is essentially a detector of synaptic activity and synaptic strength due to its influx through NMDAR and other specific channels. In the cytosol, Ca^2+^ are buffered by numerous endogenous sensors including calmodulin. The ions are also captured by storage through the SERCA pump and removed from neurons by different ionic pumps and transporters. Considering all the above-mentioned, it is not surprising that the mitochondria located near synaptic zones, or even directly within them, utilize calcium ions in different ways, but primarily as universal, high precision, fast, local, and functional regulators of ATP production.

In the present review, the potential mechanism of Ca^2+^ interaction with postsynaptic mitochondria in the context of the ion’s dynamics and its influence on ATP production was described. A few brief conclusions should be drawn:

(1) Neuronal mitochondria can be relocated from the soma to distal parts of the cell. Their functioning requires a high level of self-sufficiency and rapid reactions to energy requests. Synaptic mitochondria experience considerable functional load. This is why mitochondria requires a reliable detector for the activation of local ATP production.
(2) In dendrites, mitochondria form spatially fixed clusters. Mitochondrial distribution depends on local dendritic Ca^2+^ spikes; in other words, the distribution occurs in the dendritic compartments associated with a specific synapse. Because of the energetic requirement for local plasticity, dendrites need new mitochondria to be attracted, as well as the removal of dysfunctional ones by mitophagy.
(3) It is hypothesized that ATP synthase distribution and activity depend on postsynaptic Ca^2+^-transients. ATP synthase monomers form dimer or tetramer complexes, which arrange into linear rows forming a helical structure on cristae. This arrangement suggests the possibility of varying ATP synthase concentrations in specific mitochondrial cluster zones.
(4) There are some, albeit currently insufficient, grounds to believe that locally occurring Ca^2+^ gradients stimulate not only local but also direct ATP synthesis required for activation maintenance in the postsynaptic area (more frequently, this is a dendritic spine).
(5) Entering the mitochondrial matrix through VDAC and MCU, Ca^2+^ could modulate ATP synthase activity. For instance, the ions’ indirect influence may occur through the Krebs cycle dehydrogenases, while their direct influence could occur through ATP synthase binding to the F1 complex β-subunit. ATP secretion into the cytosol via the ATP/ADP carrier also shows evidence of Ca^2+^-dependence.

Direct measurements of ATP levels and calcium regulation of synthesis in MTs remain a challenging experimental task. The limitations arise from several factors. First, mitochondrial events typically occur in extremely small volumes, complicating any direct measurements. Second, the inner membrane of intact MTs is an insurmountable barrier for many water-soluble markers. Third, proteins such as genetically encoded calcium probes are sources of experimental artifacts during measurement. Fourth, neurons exhibit millisecond dynamics of electrical and ionic fluctuations, which requires high-speed detection, while it is difficult to combine high imaging resolution and high speed. Finally, physiological ATP levels are maintained at 1–5 mM, making it extremely difficult to measure local fluctuations of these molecules, which probably do not exceed a few percent of the baseline level. A good way out may be mathematical modeling, which does not suffer from the listed limitations. However, there has been little progress in this area so far.

Thus, the model by Pokhilko et al. (2006), describes the rapid kinetics of calcium infusion into mitochondria. The model shows that at concentrations below 140 nM, all available Ca^2+^ ions are accumulated in the matrix without permeability transition pore activation, while levels above 140 nM promote periodic or continuous pore opening and system oscillations (see Figure 9). The model by Tarraf et al. (2021) predicts the rate of ATP generation in accordance with homeostatic needs. One of the important factors through which calcium may affect ATP levels is the kinetics of the uniporter. The model proposed by Dash et al. (2009) describes the kinetics of Ca^2+^ transport through the uniporter at different ΔΨ and ion concentrations. Despite the apparent attractiveness, the large number of partly uncertain parameters limit the mathematical modeling of mitochondrial dynamics in the postsynaptic area.

Many mechanisms described in our review remain hypothetical, unproven and require further experimental verification. Thus, possible uneven distribution of ATPs, MCU and ANT in postsynaptic mitochondria requires the use of super-resolution or expansion microscopy, since conventional confocal imaging does not provide sufficient resolution. Crispr/Cas methods, the use of transgenic animals and plasmids carrying mutant genes can indicate whether the lack/malfunction of a set of key proteins, such as PSEN1/2, MCU, RyR/IP_3_R will affect ATP production. Functional dynamic experiments (electrophysiology, live imaging, glutamate uncaging) along with stimulation or suppression of local synaptic activity can best link synapse function with ATP production. An adequate correlate could be the measurement of mitochondrial potential (using tetramethylrhodamine, TMRM) or intraorganellar calcium (e.g., MT-linked CaMPs). Still, the most effective indication seems to be the direct measurement of ATP postsynaptic fluctuations using recently developed genetically encoded ATP sensors.

In conclusion, the hypothetical nature of some of our suppositions should be emphasized, and new experimental evidence is required to test these assumptions. Future research focusing on the intricacies of ATP synthase distribution, local calcium gradients, and their influence on mitochondrial processes in the postsynaptic zone could unveil new horizons in understanding the mechanisms of neuronal plasticity and energy regulation. Such knowledge will contribute to a deeper comprehension of the foundations of cognitive processes and the development of therapeutic approaches to neurodegenerative diseases.

## Figures and Tables

**Table:** 

AAC	ADP/ATP carrier
ADP	Adenosine diphosphate
AMPAR	α-amino-3-hydroxy-5-methyl-4-isoxazolepropionic acid receptors
ANT	Adenine nucleotide translocator
AP	Action potential
ATP	Adenosine triphosphate
ATPs	ATP synthase
CaM	Calmodulin
CaMKII	Ca^2+^/calmodulin-dependent protein kinase II
CICR	Calcium-induced calcium release
CRT	Calreticulin
CS	central stalk of ATP synthase
CyD	Cyclophilin D
DAG	Diacylglycerol
DJ-1	Deglycase
Drp1	GTPase Dynamin-1-like protein
eATPase	Ectopic ATPase
ER	Endoplasmic reticulum
ETC	Electron-transport chain
IF1	ATPase Inhibitory Factor 1
IMF	Intermyofibrillar
IMM	Inner mitochondrial membrane
IP_3_R	Inositol trisphosphate receptor
IP_3_	Inositol trisphosphate
GABABRs	Gamma-aminobutyric acid B receptor
GIRK	G-protein-coupled inwardly rectifying K^+^ channels
Grp75	Protein glucose-regulated protein 75
GTP	Guanosine triphosphatases
KIF5B	Kinesin family member 5B
LTD	Long-term depression
LTP	Long-term potentiation
MACP	Mitochondrial anion carrier proteins
MAM	Mitochondria-associated membranes of ER
MCU	Mitochondrial calcium uniporter
Mfn	Mitofusin
mPTP	Mitochondrial permeability transition pore
Miro 1/2	Mitochondrial Rho GTPase 1/2
MT	Mitochondria
NMDAR	N-methyl-D-aspartate receptor
NO	Nitric oxide
NOS	Nitric oxide synthase
OSCP	Oligomycin-sensitivity conferring protein
OMM	Outer mitochondrial membrane
OPA1	Dynamin-like GTPase Optic Atrophy 1
OXPHOS	Mitochondrial oxidative phosphorylation
PINK	PTEN-induced kinase 1
PIP_2_	Phosphatidylinositol 4,5-bisphosphate
PLC	Phospholipase C
PKC	Protein kinase C
PS	Peripheral stalk of ATP synthase
PSEN1/2	Presenilins 1/2
RNS	Reactive nitrogen species
ROS	Reactive oxygen species
RyR	Ryanodine receptor
SA	Spine apparatus
SCaMC	Short calcium-binding mitochondrial carrier
SERCA	Sarcoplasmic/endoplasmic reticulum Ca^2+^-ATPase
SSM	Subsarcolemmal
TCA	Tricarboxylic acid
TRAK	Trafficking kinesin protein
UCP4	Uncoupling protein
VDAC	Voltage-dependent anion channel
VGCC	Voltage-gated calcium channels
VGSC	Voltage-gated sodium channels
ΔΨmt	Mitochondrial membrane potential
[Ca^2+^]_c_	Cytosolic calcium concentration
[Ca^2+^]_m_	Mitochondrial calcium concentration
σ1R	Sigma-1 receptor
